# GSK-3β protects fetal oocytes from premature death via modulating TAp63 expression in mice

**DOI:** 10.1186/s12915-019-0641-9

**Published:** 2019-03-12

**Authors:** Jia Wen, Hao Yan, Meina He, Tuo Zhang, Xinyi Mu, Haibin Wang, Hua Zhang, Guoliang Xia, Chao Wang

**Affiliations:** 1 State Key Laboratory of Agrobiotechnology, College of Biological Sciences, China Agricultural University, Beijing, 100193 China; 2 Fujian Provincial Key Laboratory of Reproductive Health Research, Medical College of Xiamen University, Xiamen, 361005 Fujian China; 3 Key Laboratory of Ministry of Education for Conservation and Utilization of Special Biological Resources in the Western China, College of Life Science, Ningxia University, 539 W Helanshan Road, Xixia District, Yinchuan, 750021 Ningxia China

**Keywords:** DNA damage checkpoint, GSK-3β, Meiotic prophase I, Oocytes, Primordial follicle

## Abstract

**Background:**

Female mammals have a limited reproductive lifespan determined by the size of the primordial follicle pool established perinatally. Over two thirds of fetal oocytes are abolished via programmed cell death during early folliculogenesis. However, the underlying mechanisms governing fetal oocyte attrition remain largely elusive.

**Results:**

Here, we demonstrate that glycogen synthase kinase-3 beta (GSK-3β) is indispensable for fetal oocyte maintenance during meiotic prophase I in mice. In vitro inhibition of GSK-3β activity or in vivo conditional deletion of *Gsk-3β* in the germline led to a dramatic loss of fetal oocytes via apoptosis, which subsequently resulted in a reduced capacity of the primordial follicle pool. Inhibition of GSK-3β also impeded meiotic progression in fetal oocytes and led to a deficiency in DNA double-strand break (DSB) repair associated with premature upregulation of Tap63, the major genome guardian of the female germline, following GSK-3β inhibition in fetal ovaries. Mechanistically, we demonstrated that aberrant nuclear translocation of β-catenin was responsible for the abnormal expression of TAp63 and global fetal oocyte attrition following GSK-3β inhibition.

**Conclusions:**

In summary, GSK-3β was essential for sustaining fetal oocyte survival and folliculogenesis via fine-tuning the cytoplasmic-nuclear translocation of β-catenin, which in turn modulates timely TAp63 expression during meiotic prophase I in mice. Our study provides a perspective on the physiological regulatory role of DNA damage checkpoint signaling in fetal oocyte guardianship and female fertility.

**Electronic supplementary material:**

The online version of this article (10.1186/s12915-019-0641-9) contains supplementary material, which is available to authorized users.

## Background

Female mammals have a finite reproductive lifespan. Due to the lack of germline stem cells in the adult ovary, the ova resource is nonrenewable after birth, and originally generated primordial follicles are gradually exhausted. Thus, an in-depth study of fetal oocyte maintenance and attrition may help to better understand the pathogenic mechanism of primary premature ovarian insufficiency (POI) and female infertility in mammals [1].

Appropriate oogenesis and early folliculogenesis during the fetal and neonatal stage determine the lifetime reproductive capacity in a female. Primordial germ cells (PGCs) start to migrate to and colonize the genital ridge from approximately 9.5–10.5 days postcoitus (dpc) in mice (36–42 dpc in humans). These cells divide rapidly without cytokinesis, which results in the formation of germ cell cysts [2–4]. In female, at 13.5 dpc in mice (64 dpc in humans), PGCs cease mitotic division and begin to enter meiosis asynchronously [4, 5]. After undergoing four substages of meiotic prophase I, termed the leptotene, zygotene, pachytene, and diplotene stages, oocytes become arrested in the dictyate stage and subsequently interact with the surrounding pregranulosa cells in a highly orchestrated process to form individual primordial follicles [6, 7]. The establishment of the primordial follicle pool is completed around birth in mice (approximately 112 dpc in humans), and the entire oocyte reserve that a female possesses is fully determined then [8, 9]. Intriguingly, over half the number of fetal oocytes is selectively abolished perinatally, at least partially via the programmed cell death (PCD) according to previous studies [10, 11]. However, the underlying mechanism that regulates fetal oocyte sustainment and elimination is still poorly understood.

The accomplishment of meiotic prophase progression is crucial for the formation of healthy primordial follicles [12]. During meiotic prophase I, an extraordinary level of programmed DNA double-strand break (DSB) tolerance has been shown in fetal oocytes [12–14]. As a member of the p53 tumor suppressor family, p63 (specifically the TAp63 isoform) is constitutively expressed in oocytes after birth and is a conserved guardian of the female germline genome [15–17]. In the process of monitoring the genome integrity, DNA damage induces phosphorylation of TAp63 and initiates p63-dependent activation of the pro-apoptotic program in oocytes [16]. Recent studies have demonstrated that the activation of TAp63 within the oocytes is required for the chemotherapy- and radiation therapy-induced oocyte apoptosis observed during cancer therapy [16, 18], which reveals the indispensable role of TAp63 in DSB supervision and germline fidelity maintenance after birth. Nevertheless, the regulatory mechanism of TAp63 in fetal oocyte PCD before primordial follicle formation perinatally is still unclear.

Glycogen synthase kinase-3 beta (GSK-3β) is a highly conserved serine-threonine kinase, which was originally discovered as a regulator of glycogenesis. Studies have revealed that GSK-3β exerts a regulatory influence on diverse cellular processes [19], including proliferation [20], differentiation [21], and apoptosis [22]. Null mutations in *Gsk-3β* in mice result in embryonic lethality caused by severe hepatocyte apoptosis [23]. Multiple functions of GSK-3β in organisms are achieved through its constitutively active phosphorylation on multiple intercellular substrates, which encompass both cytoplasmic proteins and over a dozen transcriptional factors, such as c-Jun, CREB, and Tau protein [24, 25]. GSK-3β acts as a negative regulator of the co-transcriptional factor β-catenin in the canonical WNT (wingless-type mouse mammary tumor virus integration site family)/β-catenin signaling cascade, which is widely reported regulating the mammalian reproductive system development [26]. Studies demonstrated that GSK-3β is enriched in arrested metaphase II oocytes and correlates with spindle stability [27]. GSK-3 also plays a role in the metabolic pathway during oocyte maturation [28]. Loss of GSK3 activity in oocytes at the primary follicle stage does not alter female fertility in mice. However, the offspring that were derived from those oocytes displayed neonatal death due to cardiac hyperplasia [29]. Unfortunately, the role of GSK-3β in meiotic prophase I and early folliculogenesis during the fetal stage remains unclear in mammals. Whether GSK-3β activity serves as a prerequisite for fetal oocyte development requires comprehensive exploration.

Here, we clarified the vital function of GSK-3β in maintaining fetal ovarian development in mice. Our study demonstrated that GSK-3β was necessary for sustaining fetal oocyte survival and, subsequently, early folliculogenesis in mice. Inhibition of GSK-3β resulted in premature oocyte apoptosis, accompanied by persistent unrepaired DSBs in oocytes. The underlying mechanism revealed that inhibition of GSK-3β induced β-catenin translocation to the nucleus of the oocytes, which in turn stimulated premature TAp63 expression and initiated apoptosis during meiotic prophase I. Our results provide insight into the regulatory relationship between GSK-3β- and TAp63-induced PCD of oocytes in the fetal ovaries in mice.

## Results

### GSK-3β displayed decreased activity in fetal oocytes in mice

**Fig. 1:**
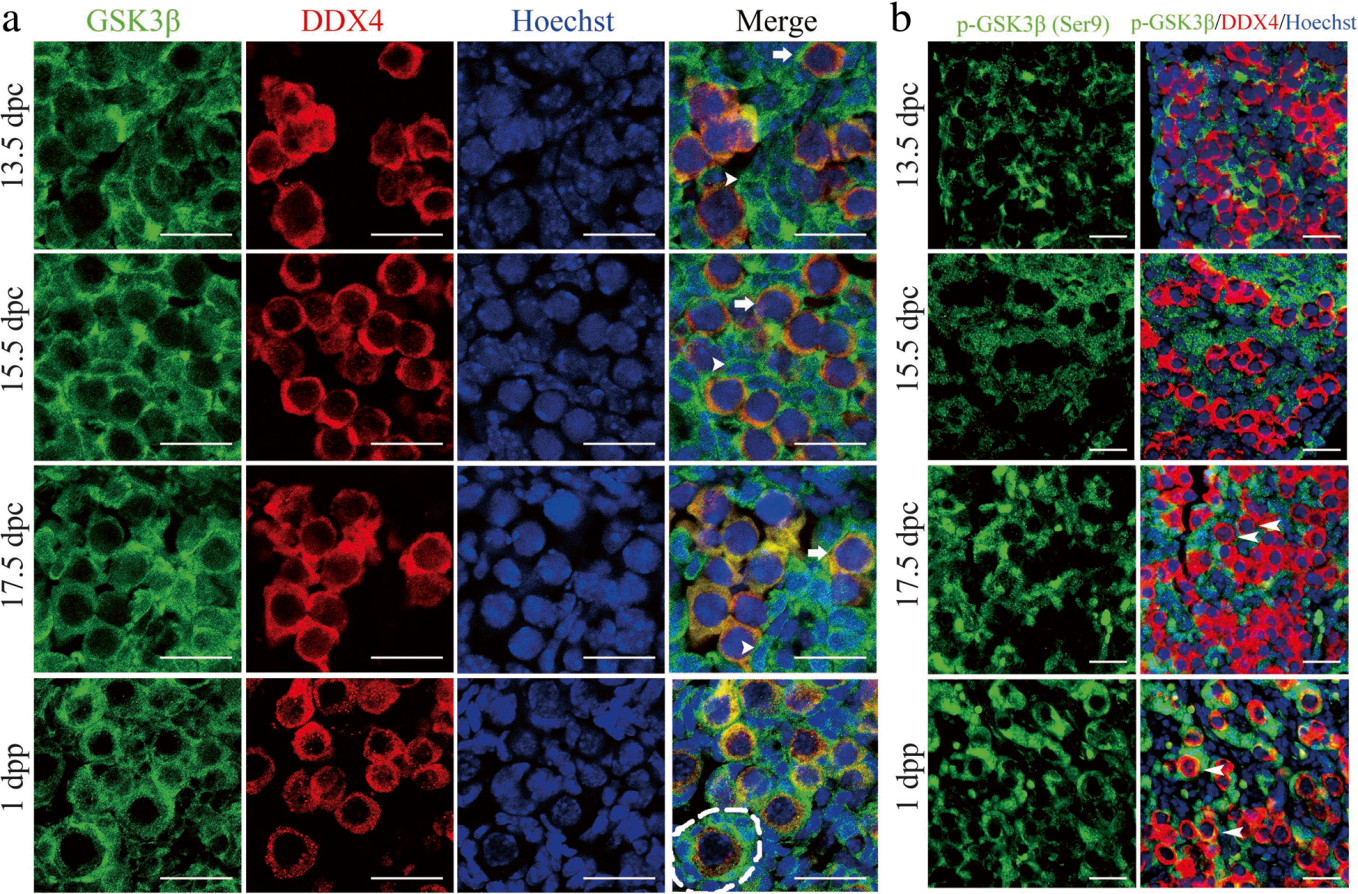
The expression patterns of GSK-3β in fetal and neonatal mouse ovaries. **a** GSK-3β localization in mouse ovaries from 13.5 dpc to 1 dpp. Mouse ovaries from 13.5 dpc, 15.5 dpc, 17.5 dpc, and 1 dpp were immunostained for GSK-3β (green) and germ cell marker DDX4 (red). The nucleus was stained by Hoechst (blue). GSK-3β was mainly expressed in the cytoplasm of both somatic cells (arrowhead) and oocytes (arrow) from 13.5 to 17.5 dpc. In the 1 dpp mouse ovary, GSK-3β was expressed in the cytoplasm of oocytes and the surrounding pre-granulosa cells of the formed primordial follicle (dashed line). **b** Phosphorylated GSK-3β at Ser9 (p-GSK-3β) was identified in mouse ovaries from 13.5 dpc to 1 dpp. The sections were immunostained for p-GSK-3β (green) and DDX4 (red). The nucleus was stained by Hoechst (blue). p-GSK-3β was scarcely expressed in oocytes before 17.5 dpc; part of the oocytes was stained for p-GSK-3β on 17.5 dpc (arrowhead), and the majority of oocytes expressed p-GSK-3β on 1 dpp (arrowhead). Scale bars, 200 μm

To explore the physiological function of GSK-3β during early oogenesis, we first examined the specific location of GSK-3β in mouse ovaries. Immunofluorescence detection showed that GSK-3β (green) was extensively expressed in mouse ovaries from 13.5 dpc to 1 dpp (days postparturition) and was primarily located within the cytoplasm of both somatic (arrowhead) and germ cells (arrow), which were marked by DEAD-Box Helicase 4 (DDX4) (red). Hoechst (blue) was used to mark the cell nucleus. On 1 dpp, when the primordial follicles began to form, GSK-3β was expressed in the cytoplasm of both oocytes and the surrounding pre-granulose cells of the primordial follicle (dashed line) (Fig. 1a). Meanwhile, we detected the expression of p-GSK-3β (Ser9), which is the inactive form of GSK-3β, in fetal and neonatal ovaries (Fig. 1b). Different from the consistent expression pattern of total GSK-3β, p-GSK-3β was almost undetectable in fetal oocytes before 17.5 dpc. Thereafter, p-GSK-3β appeared in the cytoplasm of the part of the oocytes on 17.5 dpc (arrowhead) and displayed apparent expression in most of the oocytes in 1 dpp ovaries (arrowhead). This progressively increasing expression of p-GSK-3β in fetal oocytes indicated decreasing GSK-3β activity correlating with meiotic prophase progression.

Furthermore, to determine the expression level of GSK-3β in fetal oocytes, ovarian somatic and germ cell components were separated before examination (Additional file 1: Figure S1). Western blotting results from the fetal oocyte component demonstrated that total GSK-3β protein was constantly but invariantly expressed from 13.5 dpc to 1 dpp, while p-GSK-3β expression increased significantly from 17.5 dpc onward (Additional file 1: Figure S1). The increased protein level of p-GSK-3β confirmed the downregulation of GSK-3β activity in fetal oocytes, which implied a potential functional role of GSK-3β during early meiotic prophase I in mice.

### Inhibition of GSK-3β led to dramatic fetal oocyte loss during meiotic prophase I

To thoroughly study the developmental stage-dependent role of GSK-3β in fetal ovarian development, the GSK-3β-specific inhibitor BIO (6-bromoindirubin-3′-oxime) was applied to block its activity in an in vitro culture system [30].

First, the role of GSK-3β in PGC proliferation and meiosis initiation was studied. Because the majority of PGCs switched from mitosis to meiosis at approximately 13.5 dpc in mice, 12.5 dpc ovaries were cultured for 2 days in vitro (equaling 14.5 dpc) with 1 μM dimethylsulfoxide (DMSO, as a control) or BIO. The 5-bromo-2-deoxyuridine (BrdU) labeling assay showed that proliferating PGCs (co-stained with both DDX4 antibody and BrdU antibody) were comparable to those of the control following GSK-3β inhibition (Additional file 2: Figure S2A), as was confirmed by the statistical analysis (58.00 ± 11.26 for BIO versus 55.50 ± 12.63 for the control per section; *P* > 0.05) (Additional file 2: Figure S2B). Similar results were obtained when the cultured ovaries were stained with another cellular proliferation marker, proliferating cell nuclear antigen (PCNA) (144.40 ± 33.43 for BIO versus 133.67 ± 30.73 for the control per section; *P* > 0.05) (Additional file 2: Figure S2C-D). Moreover, to examine the meiotic initiation in PGCs following GSK-3β inhibition, cultured ovaries were stained with an antibody against synaptonemal complex protein 3 (SYCP3) to mark the germ cells that had entered meiosis. The results showed that SYCP3 showed a weak appearance on 13.5 dpc but became intensively expressed in the germ cell nuclei from the leptotene stage onward (Additional file 2: Figure S2E) [31]. As shown in Additional file 2: Figure S2E, the majority of the germ cells from both the control and GSK-3β-inhibited group entered meiosis normally. Taken together, inhibition of GSK-3β had no significant impact on PGC proliferation or meiosis initiation in the fetal ovaries in mice.

**Fig. 2:**
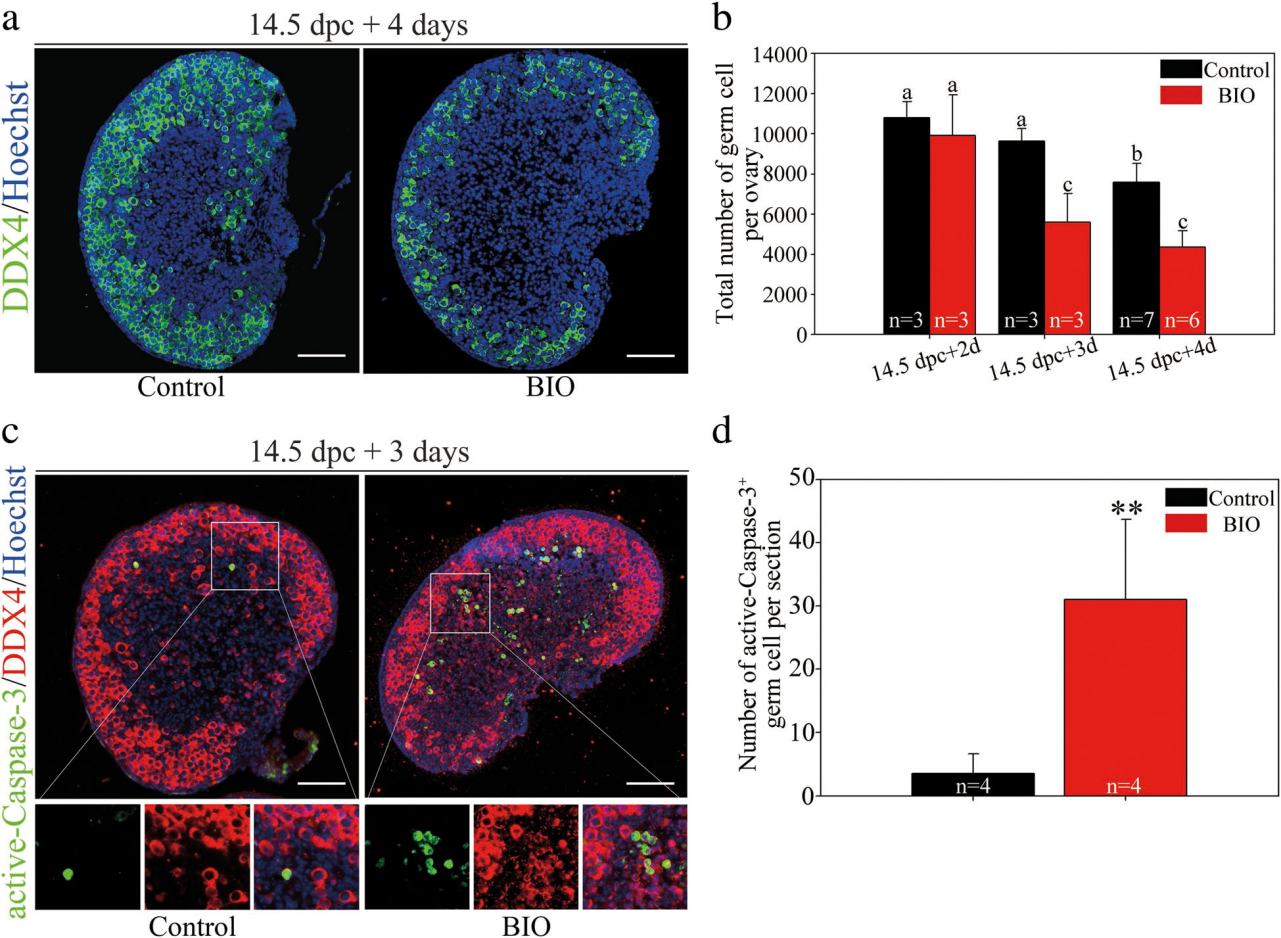
GSK-3β was indispensable for oocyte survival in fetal mice. **a**, **b** Inhibition of GSK-3β by BIO led to dramatic oocyte loss in fetal ovaries. Before the examination, ovaries at 14.5 dpc were cultured in vitro with either DMSO (as the control) or the GSK-3β-specific inhibitor BIO for 4 days. **a** Oocytes were stained with DDX4 (green). The nucleus was stained with Hoechst (blue). **b** Statistical analysis showed that the total number of oocytes decreased time-dependently following BIO treatment for 2, 3, and 4 days (Additional file 8: Individual data values). The data are presented as means ± s.d. Different letters (a–c) denote a statistically significant difference between the groups (ANOVA and Holm-Sidak test). **c**, **d** Inhibition of GSK-3β caused severe cellular apoptosis in the fetal ovaries. Before the examination, ovaries at 14.5 dpc were cultured in vitro with DMSO or BIO for 3 days. **c** Active Caspase-3 signals (green) corresponded to apoptotic cells. Oocytes were stained with DDX4 (red). The nucleus was stained by Hoechst (blue). **d** Statistical analysis showed that the number of apoptotic oocytes per section increased significantly following BIO treatment (Additional file 8: Individual data values). The data are presented as mean ± s.d. The asterisk (*) denotes a statistically significant difference between the control and treatment groups. *P < 0.05, **P < 0.01, and ***P < 0.001 (*t* test). Scale bars, 200 μm

Next, to explore the function of GSK-3β during meiotic prophase I, 14.5 dpc ovaries were cultured for 2, 3, and 4 days (equaling 16.5 dpc, 17.5 dpc, and 18.5 dpc, respectively) with BIO. Immunofluorescence examination revealed that the oocyte quantity declined dramatically in GSK-3β-inhibited ovaries compared with that in the control after 4 days of culture (Fig. 2a). The statistical analysis revealed that the oocyte number decreased in a time-dependent manner after GSK-3β inhibition and that GSK-3β inhibition resulted in the ovaries containing approximately 50% fewer oocytes (4370.83 ± 790.14) than the control ovaries (7580.00 ± 964.40) (*P* < 0.001) after 4 days of treatment (Fig. 2b). To confirm the role of GSK-3β in fetal oocyte conservation in the mouse ovary, another GSK-3β-specific inhibitor, CHIR99021 (5 μM), was used. The results of the immunofluorescence (Additional file 3: Figure S3A) and statistical analysis (Additional file 3: Figure S3B) verified consistent oocyte loss (4183.75 ± 667.85 for CHIR99021 versus 7805.00 ± 961.99 for the control per ovary; *P* < 0.001) following 4 days of GSK-3β inhibition. Moreover, after 14.5 dpc ovaries were cultured with BIO for 3 days (equaling 17.5 dpc), we found apparently severe oocyte apoptosis according to the immunofluorescence co-staining of DDX4 and active Caspase-3, which has been proven to be involved in programmed oocyte apoptosis in the fetal ovary [32] (Fig. 2c). Statistical analysis confirmed the significantly increased oocyte apoptosis after GSK-3β inhibition in the fetal ovary (31.00 ± 12.68 for BIO versus 3.50 ± 3.11 for the control per section; *P* < 0.01) (Fig. 2d). In addition, western blotting results showed that the protein level of Caspase-3 in the fetal ovaries increased significantly following GSK-3β inhibition (Additional file 3: Figure S3C). These results implied that GSK-3β was indispensable for fetal oocyte survival during meiotic prophase I, as inhibition of GSK-3β in the fetal ovaries led to massive oocyte apoptosis.

Finally, to determine the function of GSK-3β during germline cyst breakdown and primordial follicle formation, 17.5 dpc ovaries were cultured with BIO for 4 days (equaling 2 dpp). Immunofluorescence results showed that primordial follicle assembly (arrowhead) was intact following GSK-3β inhibition (Additional file 3: Figure S3D). The numbers of total oocytes and the established primordial follicle showed insignificant differences between the control and GSK-3β inhibition groups (Additional file 3: Figure S3E). In addition, after 14.5 dpc ovaries were cultured with BIO for 7 days (equaling 3 dpp), the treated ovaries contained significantly fewer primordial follicle compared to control (Additional file 3: Figure S3F-G), which implied that inhibition of GSK-3β not only influenced fetal oocyte survival but also impaired further folliculogenesis.

### Inhibition of GSK-3β impeded meiotic progression and resulted in meiotic defects

**Fig. 3:**
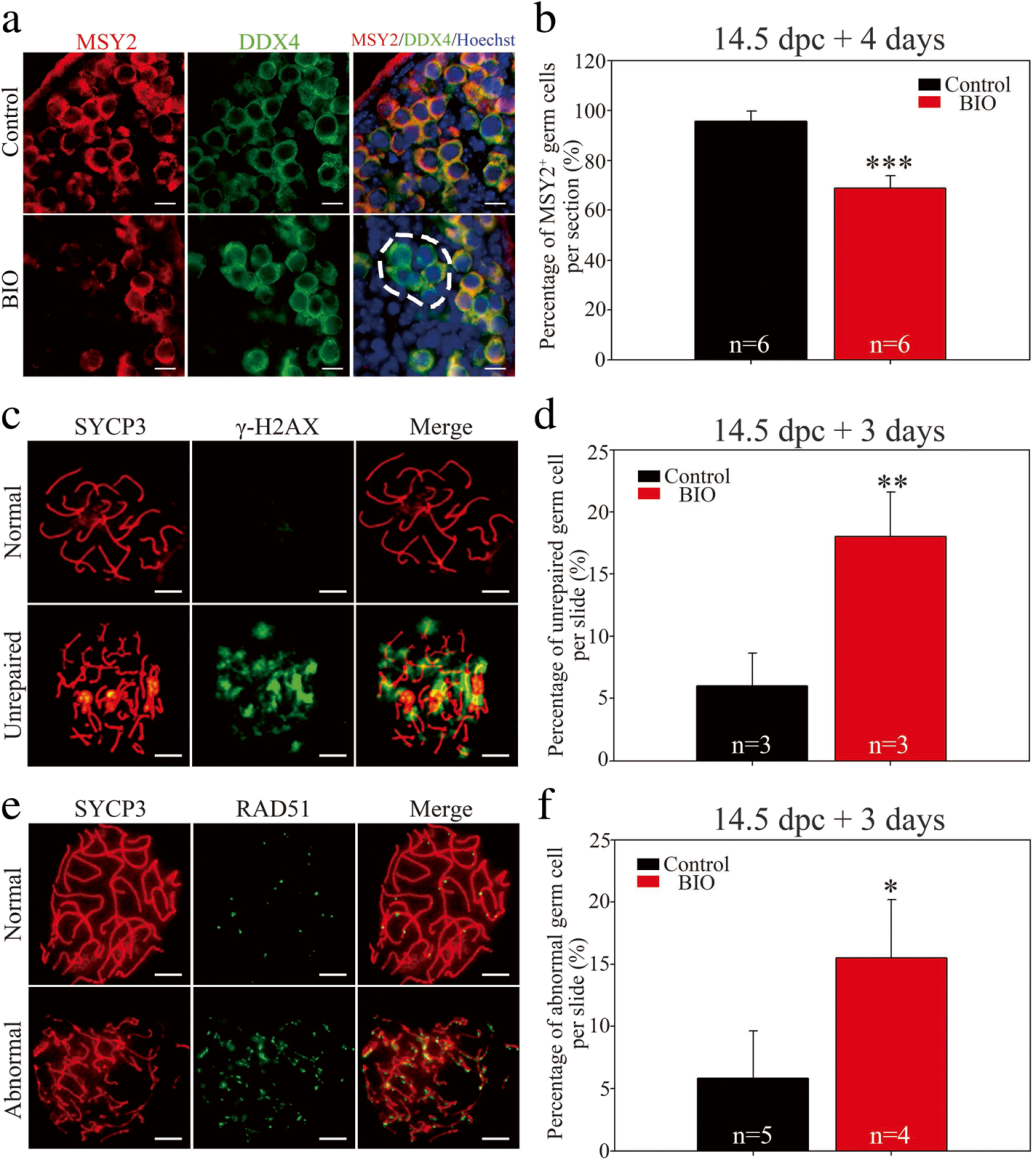
Inhibition of GSK-3β impeded meiotic progression and caused DSB repair deficiency in fetal mouse ovaries. **a**, **b** Oocytes at the diplotene stage were reduced following GSK-3β inhibition. Before the examination, ovaries at 14.5 dpc were cultured in vitro with DMSO or BIO for 4 days. **a** MSY2 (red) marked oocytes entering the diplotene stage, and DDX4 (green) marked germ cells. The nucleus was stained by Hoechst (blue). Oocytes lacking MSY2 staining are indicated by the dashed line. Scale bars, 200 μm. **b** Statistical analysis showed that the percentage of MSY2^+^ oocytes (number of cells both MSY2^+^ and DDX4^+^/number of cells DDX4^+^) per section decreased significantly following GSK-3β inhibition (Additional file 8: Individual data values). **c**, **d** Inhibition of GSK-3β caused meiotic DSB repair deficiency. Before the examination, ovaries at 14.5 dpc were cultured in vitro with DMSO or BIO for 3 days. **c** Representative images of the meiotic spread of the pachytene-stage oocytes with repaired or unrepaired DSBs. γ-H2AX (green) indicates unrepaired DSB sites. SYCP3 (red) demonstrates lateral elements. Scale bars, 10 μm. **d** Statistical analysis showed that the percentage of oocytes with unrepaired DSBs in the pachytene stage on chromosomes per slide increased significantly following GSK-3β inhibition (Additional file 8: Individual data values). **e**, **f** Inhibition of GSK-3β resulted in ectopic RAD51 expression. Before the examination, ovaries at 14.5 dpc were cultured in vitro with DMSO or BIO for 3 days. **e** Representative images of the meiotic spread of the pachytene-stage oocytes with normal or ectopic RAD51 foci. Oocyte chromosomes were co-stained with RAD51 (green) and SYCP3 (red). Scale bars, 10 μm. **f** Statistical analysis showed that the percentage of oocytes with ectopic RAD51 foci on chromosomes in the pachytene stage per slide increased significantly following GSK-3β inhibition (Additional file 8: Individual data values). The data are presented as mean ± s.d. The asterisk (*) denotes a statistically significant difference between the control and treatment groups. *P < 0.05, **P < 0.01, and ***P < 0.001 (*t* test)

Based on previous results that GSK-3β was essential for maintaining fetal oocyte survival during meiotic prophase I, we next assessed meiotic progression and meiotic events following GSK-3β inhibition. Histological immunofluorescence with antibodies against Y box protein 2 (MSY2), which is present exclusively from the diplotene stage and afterward in oocytes [33, 34], demonstrated that when 14.5 dpc ovaries were cultured for 4 days (equaling 18.5 dpc) with BIO, MSY2-positive oocytes were obviously reduced (dashed line; Fig. 3a). The statistic analysis verified that the vast majority (95.72% ± 4.02% per section) of the oocytes were MSY2^+^ in the control group, whereas significantly less (68.84% ± 4.9% per section; *P* < 0.001) oocytes from the BIO-treated group were MSY2^+^ (Fig. 3b), which implied that fetal oocytes were impeded in reaching the diplotene stage following GSK-3β inhibition.

Since partial fetal oocytes in the GSK-3β-inhibited ovaries failed to reach the diplotene stage, when programmed DSB repair was completed and synapsis was achieved, we next assessed whether GSK-3β inhibition impacted synaptic events in oocytes. Phosphorylated histone H2AX at Ser139 (referred to as γ-H2AX), which marked DSBs on meiotic chromatin, appeared in the oocytes from the leptotene stage and disappeared markedly in the pachytene stage (Additional file 4: Figure S4A) [35]. However, the chromosome spreads showed that a substantial number of fetal oocytes exhibited distinct γ-H2AX signals in the late pachytene stage following GSK-3β inhibition, indicating unrepaired DSBs sustained on the chromosomes; in contrast, normal fetal oocytes that reached the late pachytene stage were devoid of γ-H2AX signals on the chromosomes (Fig. 3c). Statistical analysis revealed that the percentage of oocytes with unrepaired DSBs in the pachytene stage was increased significantly following GSK-3β inhibition (18.00% ± 3.61% for BIO versus 6.00% ± 2.65% for the control per slide; *P* < 0.01) (Fig. 3d), which might be the reason for the failure to progress to the diplotene stage after GSK-3β inhibition. Moreover, immunofluorescence co-staining for RAD51 recombinase (a RecA homolog; a key factor in homologous recombination repair) and SYCP3 demonstrated that partial fetal oocytes showed ectopic RAD51 foci on the pachytene chromosome following GSK-3β inhibition, which implied incomplete DSB repair in the oocytes in GSK-3β-inhibited ovaries (Fig. 3e). Similarly, statistical analysis demonstrated that the percentage of abnormal RAD51-persistent oocytes in the pachytene stage in GSK-3β-inhibited ovaries (15.50% ± 4.65% per slide) increased significantly than that in the control ovaries (5.80% ± 3.83% per slide; *P* < 0.05) (Fig 3f). In summary, GSK-3β ensured the normal process of meiotic prophase I in fetal oocytes, whereas inhibition of GSK-3β resulted in abnormal meiotic DSB repair and meiotic progression errors.

### Premature TAp63 upregulation was evident during meiotic prophase I following GSK-3β inhibition

Since there was a deficiency in DSB repair, cell cycle arrest, and increased apoptosis in fetal oocytes following GSK-3β inhibition, DNA damage checkpoint signaling was presumed to be impaired in these fetal oocytes.

**Fig. 4:**
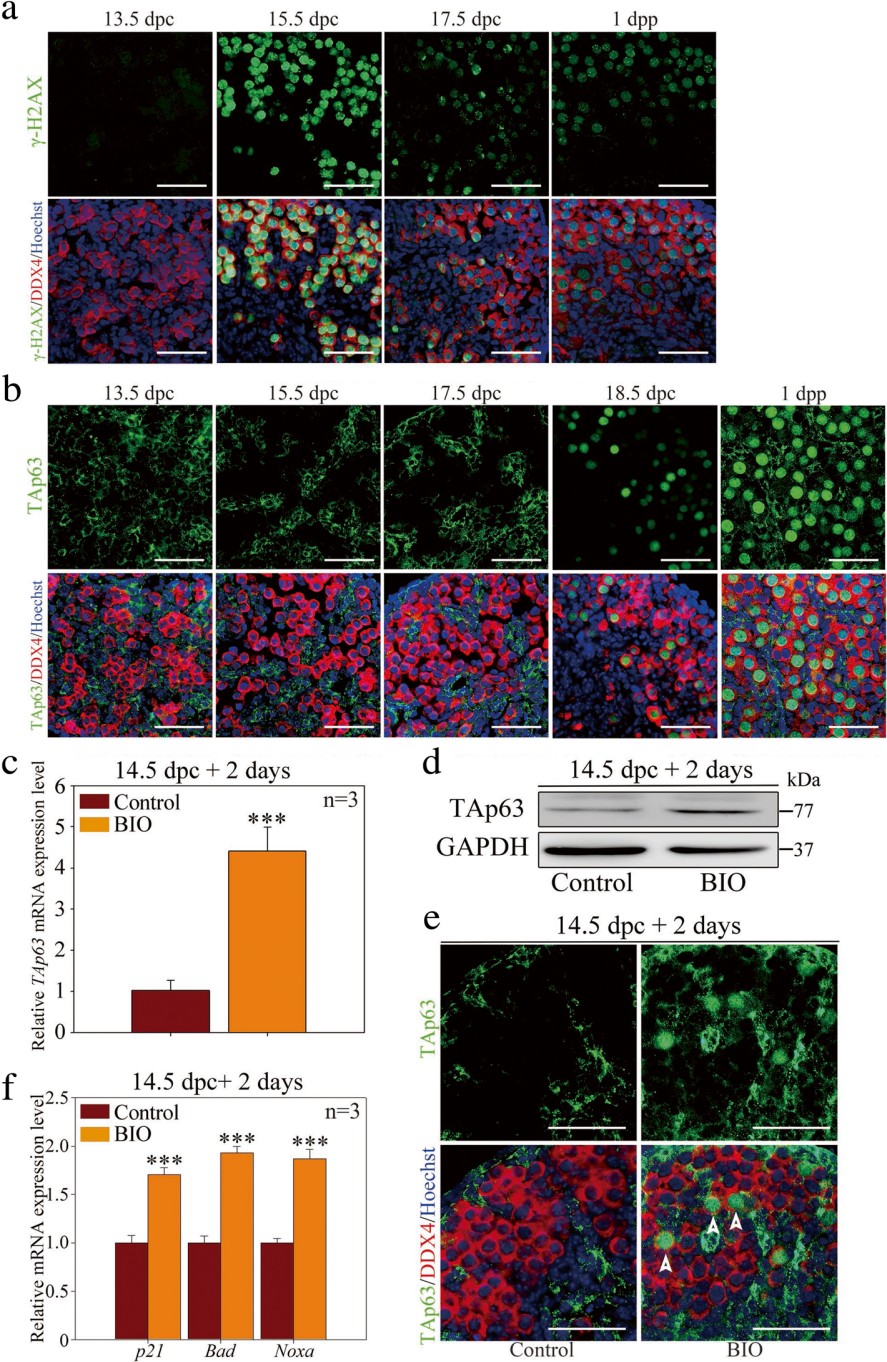
Premature upregulation of TAp63 in GSK-3β-inhibited ovary. **a** The expression pattern of γ-H2AX in fetal and neonatal mouse ovary in vivo. Mouse ovaries from 13.5 dpc, 15.5 dpc, 17.5 dpc, and 1 dpp were immunostained for γ-H2AX (green) and DDX4 (red). The nucleus was stained by Hoechst (blue). γ-H2AX displayed intensive expression in the germ cell nucleus from 15.5 to 17.5 dpc. **b** The expression pattern of TAp63 in fetal and neonatal mouse ovary in vivo. Mouse ovaries from 13.5 dpc, 15.5 dpc, 17.5 dpc, 18.5 dpc, and 1 dpp were immunostained for TAp63 (green) and DDX4 (red). The nucleus was stained by Hoechst (blue). The TAp63 protein located within somatic cells in fetal ovary and began to express in the germ cell nucleus from 18.5 dpc afterward. **c**–**e** TAp63 expression was upregulated in fetal ovary and displayed premature localization within the oocyte nucleus following GSK-3β inhibition. Before the examination, ovaries of 14.5 dpc were cultured in vitro with DMSO or BIO for 3 days. **c** qRT-PCR analysis of *TAp63* mRNA level following GSK-3β inhibition (normalized to *β-actin*) (Additional file 8: Individual data values). **d** Western blotting analysis of TAp63 protein level following GSK-3β inhibition. GAPDH was used as an internal control. **e** Immunofluorescence staining for TAp63 (green) and DDX4 (red). The nucleus was stained by Hoechst (blue). TAp63 showed premature expression within the oocyte nucleus following GSK-3β inhibition (arrowhead). **f** qRT-PCR results showed that relative mRNA expression level of *p21*, *Bad*, and *Noxa* increased significantly in GSK-3β-inhibiting ovaries (Additional file 8: Individual data values). The data are presented as mean ± s.d. The asterisk (*) denotes a statistically significant difference between the control and treatment groups. *P < 0.05, **P < 0.01, and ***P < 0.001 (*t* test). Scale bars, 200 μm

To investigate the validity of the DNA damage checkpoint signaling during meiotic prophase I, we first examined the expression pattern of key components of the signaling in fetal and neonatal ovaries in vivo. Histological sections and immunofluorescence staining showed that γ-H2AX signaling, which marked unprocessed DSBs on chromosomes, emerged intensively in the oocyte nucleus from 15.5 to 17.5 dpc and disappeared afterward (Fig. 4a). This expression pattern was correlated with meiotic prophase progression, as most DSBs were induced in the leptotene stage and accomplished recombinational repair in the late pachytene stage [36]. Accordingly, p-ATM (phosphorylated ataxia telangiectasia mutated kinase) showed notable expression from 15.5 to 17.5 dpc and was primarily located within the oocyte nucleus (Additional file 4: Figure S4B). Consistently, p-CHK2 (phosphorylated checkpoint kinase 2) showed similar expression peaks to γ-H2AX in the fetal ovary (Additional file 4: Figure S4C). Intriguingly, as the major downstream effecter that is required for culling the oocytes bearing unrepaired DSBs, TAp63 was completely absent in fetal oocytes; instead, it showed a peak in the perinatal oocyte nucleus. TAp63 displayed strong expression within the oocyte nucleus until 1 dpp, when most oocytes had reached the diplotene stage (Fig. 4b). Together, these results showed that fetal oocytes were devoid of TAp63 expression until DSB repair completion around birth in vivo.

However, when 14.5 dpc ovaries were cultured for 2 days (equaling 16.5 dpc) with BIO, both the mRNA and protein level of TAp63 increased significantly, which suggested an unexpected enhancement of TAp63 expression during meiotic prophase I (Fig. 4c, d). Correspondingly, TAp63 was found to be prematurely expressed within the nucleus of partial oocytes in the fetal ovary following GSK-3β inhibition (Fig. 4e). These findings indicated that inhibition of GSK-3β resulted in the disrupted expression pattern of TAp63 in fetal oocytes in mouse ovaries.

Furthermore, we detected the expression of downstream apoptotic inducers, *p21* (cyclin-dependent kinase inhibitor), *Bad*, *Noxa*, and *Puma*, which are BH3-only proapoptotic Bcl-2 family members and are essential mediators of p63-dependent apoptosis pathways [37]. Qualitative reverse transcription polymerase chain reaction (qRT-PCR) results showed that the relative mRNA expression levels of *p21*, *Bad*, and *Noxa* were significantly increased in GSK-3β-inhibited ovaries (Fig. 4f), which indicates p63-dependent apoptotic activation. *Puma* was rarely detectable in both the control and treatment ovaries (data not shown). In summary, inhibition of GSK-3β resulted in premature TAp63 expression and triggered transcriptions of proapoptotic genes, which might induce fetal oocyte attrition.

### GSK-3β regulated cytoplasmic-nuclear translocation of β-catenin in fetal ovaries

Since GSK-3β is pivotal for the survival of fetal oocytes, which in turn influences further folliculogenesis, it is necessary to explore the mediators downstream of GSK-3β in oocyte fate determination. According to previous reports, GSK-3β negatively regulates the canonical WNT signaling pathway via modulating the stabilization of β-catenin, an active co-transcriptional factor in the cell nucleus. Thus, the expression of β-catenin following GSK-3β inhibition was studied in fetal ovaries.

**Fig. 5:**
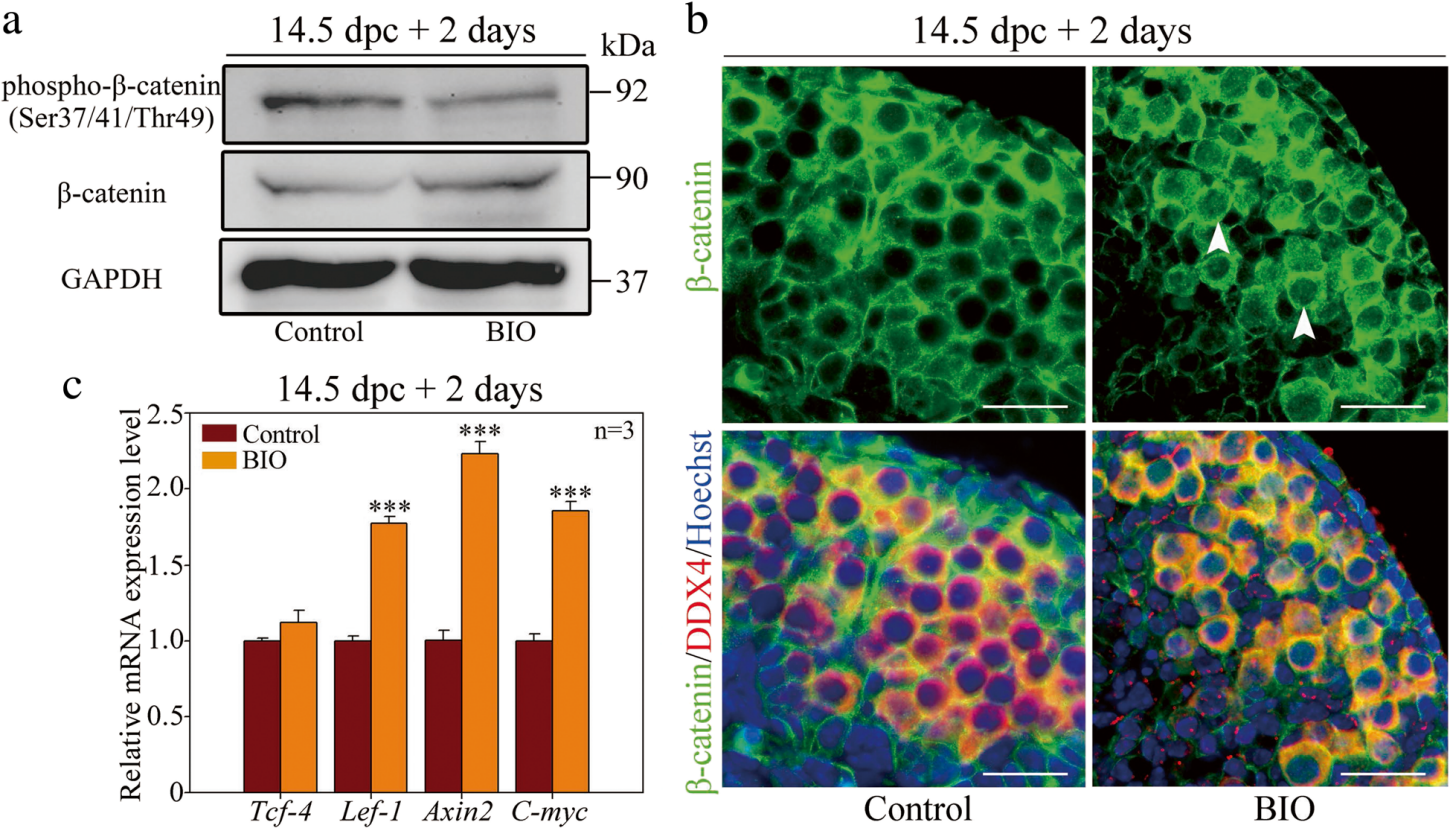
GSK-3β regulated nuclear translocation and transcriptional activation of β-catenin in the fetal ovary. **a** Inhibition of GSK-3β impaired phosphorylation of β-catenin in the fetal ovary. Western blotting showed a significant decrease in the phospho-β-catenin protein level following GSK-3β inhibition. GAPDH was used as an internal control. **b** β-catenin accumulated in the cytoplasm and translocated to the nucleus following GSK-3β inhibition. Immunofluorescence staining for β-catenin (green) and DDX4 (red). The nucleus was stained by Hoechst (blue). β-catenin displayed cytoplasmic accumulation and expression in the oocyte nucleus (arrowhead) following GSK-3β inhibition. **c** qRT-PCR analysis showed that mRNA levels of several canonical target genes of β-catenin (normalized to *β-actin*) increased significantly following GSK-3β inhibition (Additional file 8: Individual data values). The data are presented as mean ± s.d. The asterisk (*) denotes a statistically significant difference between the control and treatment groups. *P < 0.05, **P < 0.01, and ***P < 0.001 (*t* test). Scale bars, 200 μm

First, western blotting results revealed that following GSK-3β inhibition, the phospho-β-catenin (Ser37/41/Thr49) expression level decreased significantly in the fetal ovary, which implied attenuated β-catenin phosphorylation mediated by GSK-3β activity (Fig. 5a). Histological sections and immunofluorescence assays provided additional evidence that inhibition of GSK-3β significantly promoted both the cytoplasmic and nuclear staining of β-catenin in oocytes (arrowhead) compared with that in the control (Fig. 5b). Furthermore, to clarify whether the cytoplasmic accumulation of β-catenin resulted in its nuclear translocation and transcriptional activation, several canonical target genes of β-catenin were examined by qRT-PCR (Fig. 5c). The results showed that the genes were significantly upregulated following GSK-3β inhibition, which confirmed the nuclear importation of β-catenin as a co-transcriptional factor to initiate target gene transcription. In summary, inhibition of GSK-3β resulted in aberrant cytoplasmic accumulation and subsequent nuclear translocation of β-catenin in fetal oocytes.

### Aberrant nuclear translocation of β-catenin induced fetal oocyte attrition

Since GSK-3β inhibition in fetal ovaries led to dramatic fetal oocyte loss and aberrant nuclear translocation of β-catenin, accumulation and nuclear translocation of β-catenin may be responsible for the fetal oocyte attrition following GSK-3β inhibition. To evaluate this assumption, ICG-001, a specific antagonist of β-catenin-mediated transcription [38], was used to examine the presumptive role of β-catenin-mediated transcriptional activation following GSK-3β inhibition.

**Fig. 6:**
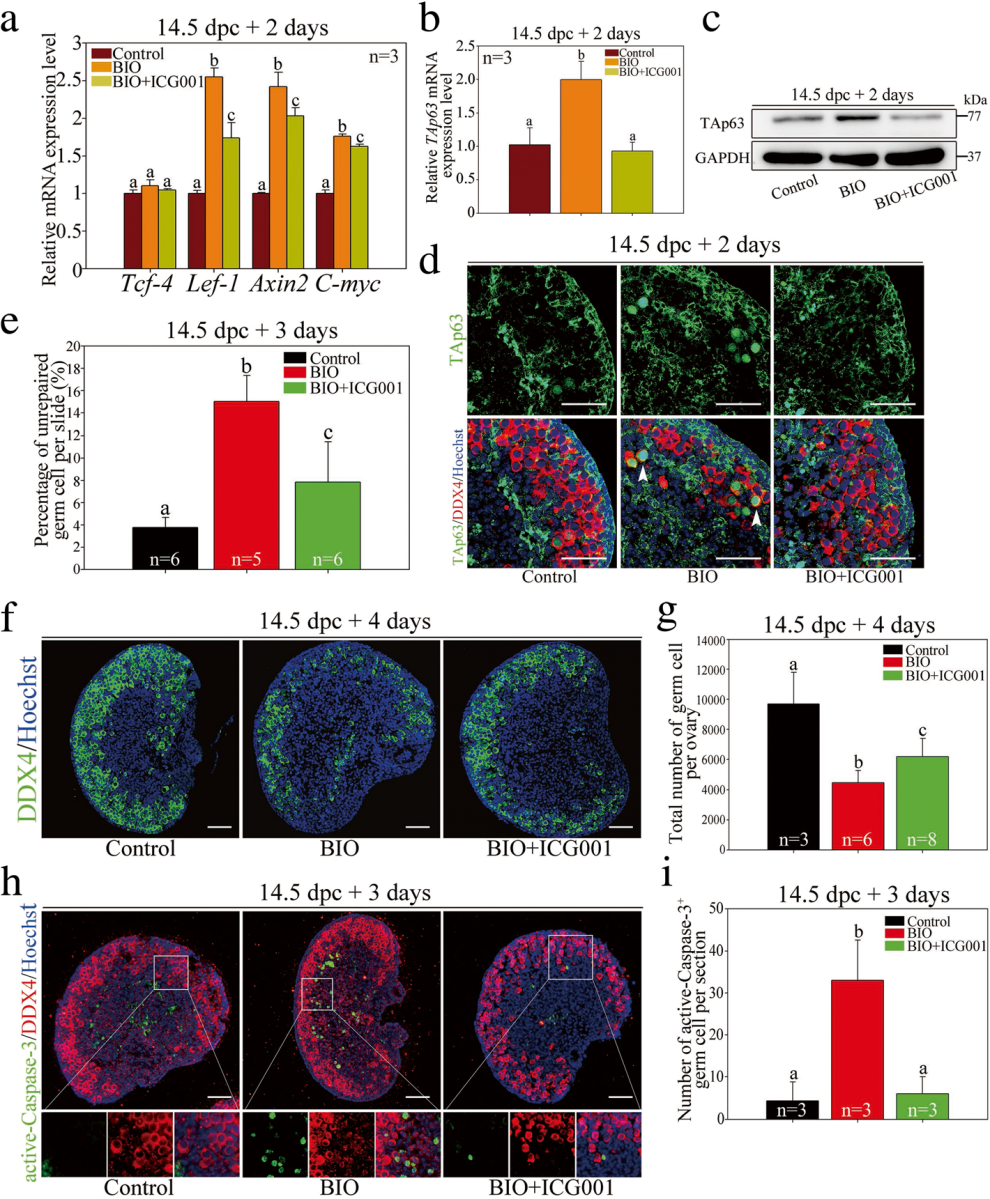
Nuclear translocation and transcriptional activation of β-catenin were detrimental to fetal oocytes. **a** qRT-PCR analysis showed that mRNA levels of target genes of β-catenin (normalized to *β-actin*) significantly decreased following combined (BIO+ICG-001) treatment compared with single (BIO) treatment (Additional file 8: Individual data values). **b**–**d** Nuclear translocation and transcriptional activation of β-catenin resulted in premature upregulation of TAp63 in the fetal ovary. Before the examination, ovaries at 14.5 dpc were cultured in vitro with DMSO or single or combined inhibitors for 2 days. **b** qRT-PCR analysis of mRNA levels of *TAp63* (normalized to *β-actin*) (Additional file 8: Individual data values). **c** Western blotting analysis of TAp63 protein levels. GAPDH was used as an internal control. **d** Immunofluorescence staining for TAp63 (green) and DDX4 (red). The nucleus was stained by Hoechst (blue). **e** Blockage of β-catenin transcriptional activity rescued DSB repair deficiency in oocytes following GSK-3β inhibition. Before the examination, ovaries at 14.5 dpc were cultured in vitro with DMSO or single or combined inhibitors for 3 days. Statistical analysis showed that the percentage of oocytes with persistent γ-H2AX signals in the pachytene stage on chromosomes per slide increased significantly following GSK-3β inhibition but reduced significantly following combined treatment (Additional file 8: Individual data values). **f**, **g** Blockage of β-catenin transcriptional activity rescued fetal oocyte loss following GSK-3β inhibition. Before the examination, ovaries at 14.5 dpc were cultured in vitro with DMSO or single or combined inhibitors for 4 days. **f** Oocytes were stained with DDX4 (green). The nucleus was stained by Hoechst (blue). **g** Statistical analysis showed that the total number of oocytes decreased significantly following a single treatment but could be rescued efficiently following combined treatment (Additional file 8: Individual data values). **h**, **i** Blockage of β-catenin transcriptional activity alleviated oocyte apoptosis following GSK-3β inhibition. Before the examination, ovaries at 14.5 dpc were cultured in vitro with DMSO single or combined inhibitors for 3 days. **h** Active Caspase-3 signals (green) corresponded to apoptotic cells. Oocytes were stained with DDX4 (red). The nucleus was stained by Hoechst (blue). **i** Statistical analysis showed that the number of apoptotic oocytes per section increased significantly following single treatment but decreased following combined treatment (Additional file 8: Individual data values). The data are presented as means ± s.d. Different letters (a–c) denote a statistically significant difference between the groups (ANOVA and Holm-Sidak test). Scale bars, 200 μm

As shown in Fig. 6a, GSK-3β inhibition significantly increased the mRNA expression levels of canonical β-catenin target genes; however, co-treatment with ICG-001 (0.5 μM) efficiently reduced the expression levels of these genes accordingly, which is indicative of the antagonizing effect of ICG-001 on β-catenin transcription activity. Importantly, the upregulated TAp63 level following BIO treatment was effectively attenuated by BIO plus ICG-001 treatment. As shown in Fig. 6b, c, when 14.5 dpc ovaries were cultured for 2 days (equaling 16.5 dpc), both the mRNA and protein levels of TAp63 increased significantly after GSK-3β inhibition but were reduced after the simultaneous block of β-catenin transcriptional activity. Meanwhile, immunofluorescence assays proved that fetal oocytes displayed aberrant nuclear TAp63 expression after GSK-3β inhibition but were devoid of nuclear TAp63 expression significantly following BIO plus ICG-001 co-treatment (Fig. 6d). These results demonstrated that by modulating the nuclear translocation of β-catenin, GSK-3β was responsible for the fine-tuning of TAp63 expression in the fetal ovary.

Next, the effect of aberrant β-catenin transcriptional activation on fetal oocyte survival was examined. Interestingly, the DSB repair deficiency in fetal oocytes following GSK-3β inhibition could be efficiently reduced when the transcription activity of β-catenin was blocked simultaneously. The percentage of the pachytene-stage oocytes with γ-H2AX signals, which indicated abnormal meiotic DSB repair, increased significantly following GSK-3β inhibition (15.04% ± 2.35% BIO versus 3.76% ± 0.93% for the control per slide; *P* < 0.05), but reduced significantly in the ovaries treated with BIO plus ICG-001 (7.86% ± 3.62% for BIO plus ICG-001 versus 15.04% ± 2.35% for BIO per slide; *P* < 0.05) (Fig. 6d).

Moreover, massive oocyte loss induced by GSK-3β inhibition was partially rescued in BIO plus ICG-001-treated ovaries (Fig. 6f). Statistical analysis confirmed a sharp decrease in oocyte quantity following GSK-3β inhibition (4468.33 ± 781.62 for BIO versus 9691.67 ± 2116.50 for the control per ovary; *P* < 0.05), whereas oocyte quantity was significantly rescued following co-treatment with BIO plus ICG-001 (6181.25 ± 1221.00 for BIO plus ICG-001 versus 4468.33 ± 781.62 for BIO per ovary; *P* < 0.05) (Fig. 6g). Accordingly, immunofluorescence co-staining of DDX4 and active Caspase-3 demonstrated that fetal oocyte apoptosis was significantly alleviated following blockage of β-catenin transcription activity (Fig. 6h). The statistical analysis revealed that the number of active Caspase-3-positive oocytes significantly increased from 4.33 ± 4.51 to 33.00 ± 9.54 per section (*P* < 0.05) following GSK-3β inhibition but decreased to 6.00 ± 4.00 per section (*P* < 0.05) when BIO plus ICG-001 treatment was applied, which was insignificantly different from the control group (*P* > 0.05) (Fig. 6i). Collectively, these results implied that following GSK-3β inhibition, nuclear translocation and transcriptional activity of β-catenin were detrimental to oocytes in the fetal ovary due to the transcriptional activation of *TAp63* expression.

### β-catenin regulated *TAp63* transcription in mouse ovaries in vivo

**Fig. 7:**
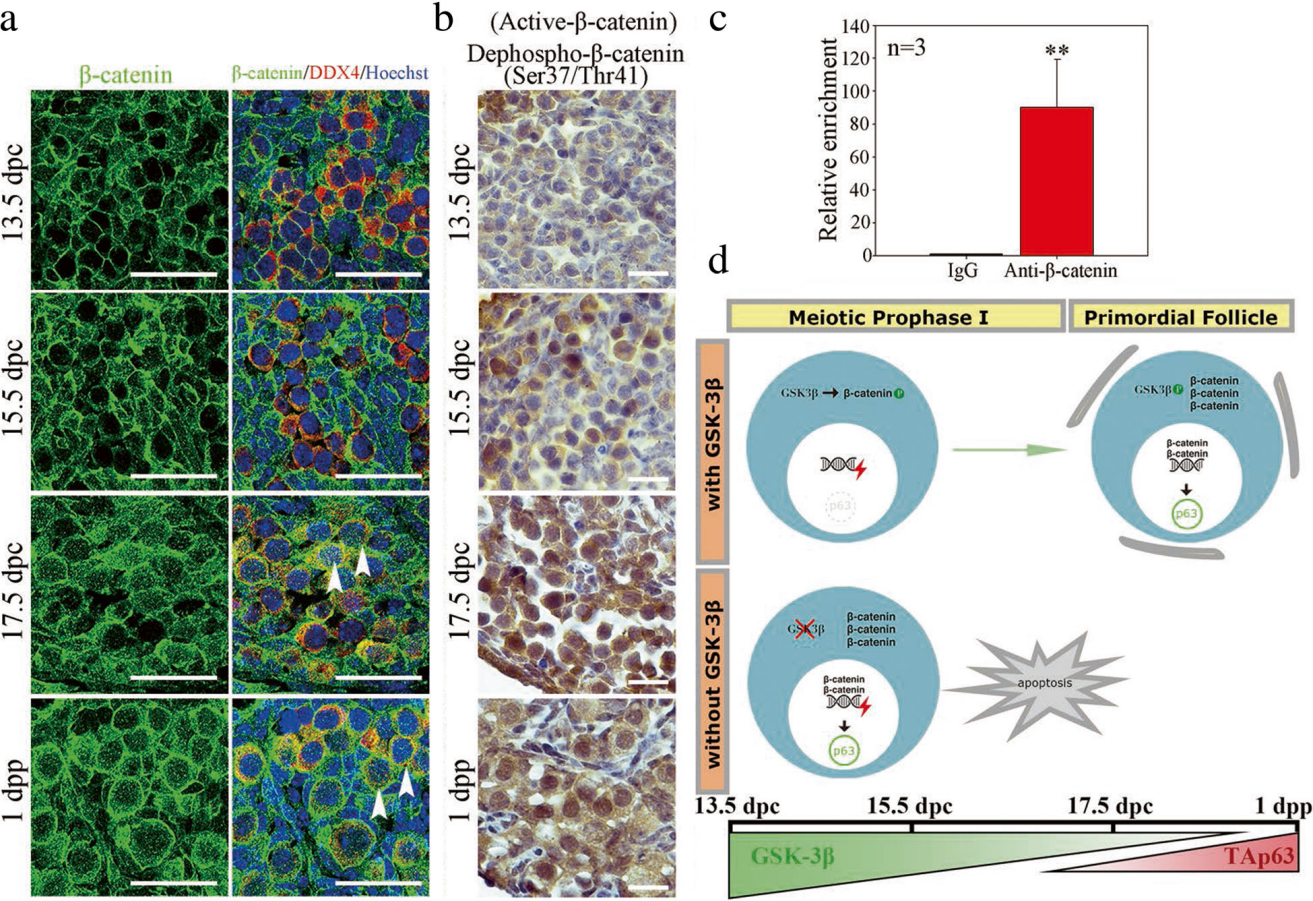
β-catenin induced *TAp63* transcriptional activation in vivo. **a** The expression of β-catenin in fetal and neonatal mouse ovary in vivo. Mouse ovaries from 13.5 dpc, 15.5 dpc, 17.5 dpc, and 1 dpp were immunostained for β-catenin (green) and DDX4 (red). The nucleus was stained by Hoechst (blue). β-catenin became intensively accumulated in the oocyte cytoplasm and nucleus from 17.5 dpc onward. **b** The expression of active β-catenin in fetal and neonatal mouse ovary in vivo. Active β-catenin began to express in the majority of the oocyte nucleus from 17.5 dpc onward. **c** ChIP-qPCR analysis of the interaction between β-catenin and *TAp63* promoters sequences in 17.5 dpc mouse ovaries (Additional file 8: Individual data values). **d** Schematic model depicting the essential role of GSK-3β in maintaining fetal oocyte survival during meiotic prophase I. The data are presented as mean ± s.d. The asterisk (*) denotes a statistically significant difference between the control and treatment groups. *P < 0.05, **P < 0.01, and ***P < 0.001 (*t* test). Scale bars, 200 μm

The regulatory relationship between β-catenin and TAp63 was then assessed in the mouse ovary in vivo. Previous studies reported that β-catenin acts as a protein with dual functions: as an intracellular adhesion on the cytomembrane and as a co-transcriptional factor in the cell nucleus [39]. According to the immunofluorescence results (Fig. 7a), β-catenin was primarily expressed on the cytomembrane of fetal oocytes before 15.5 dpc; from 17.5 dpc onward, β-catenin displayed obvious accumulation in the cytoplasm and nucleus of oocytes (arrowhead), which was correlated with the contemporaneously attenuated phosphorylation activity of GSK-3β according to our results. In addition, we detected the expression of active β-catenin, which is unphosphorylated on Ser37 or Thr41 but functionally active, in fetal and neonatal ovaries. Before 15.5 dpc, few fetal oocytes showed active β-catenin expression; however, the oocyte nucleus began to display intensive active β-catenin staining from 17.5 dpc onward (Fig. 7b). Collectively, β-catenin showed increasing nuclear expression along with meiotic prophase I progression, which was consistent with the upregulated expression of *TAp63* in fetal ovaries (Additional file 4: Figure S4D). These results implied a transcriptional regulatory role of β-catenin on *TAp63*.

Furthermore, chromatin immunoprecipitation (ChIP) assays were applied to verify whether β-catenin induced *TAp63* transcription activation directly in fetal ovaries. Mouse ovaries of 17.5 dpc were collected, and the DNA fragment that immunoprecipitated with the anti-β-catenin antibody was examined with primers designed within 2000 bp of the *TAp63* promoter sequence region. The qRT-PCR results indicated that β-catenin enriched the − 963 to − 793 region of the *TAp63* promoter sequence (Fig. 7c). In summary, these findings demonstrated that β-catenin translocated to the nucleus of fetal oocytes and activated *TAp63* transcription by directly binding to the promoter region of *TAp63* in vivo.

### Conditional deletion of *Gsk-3β* in germline cells caused oocyte loss in mice

**Fig. 8:**
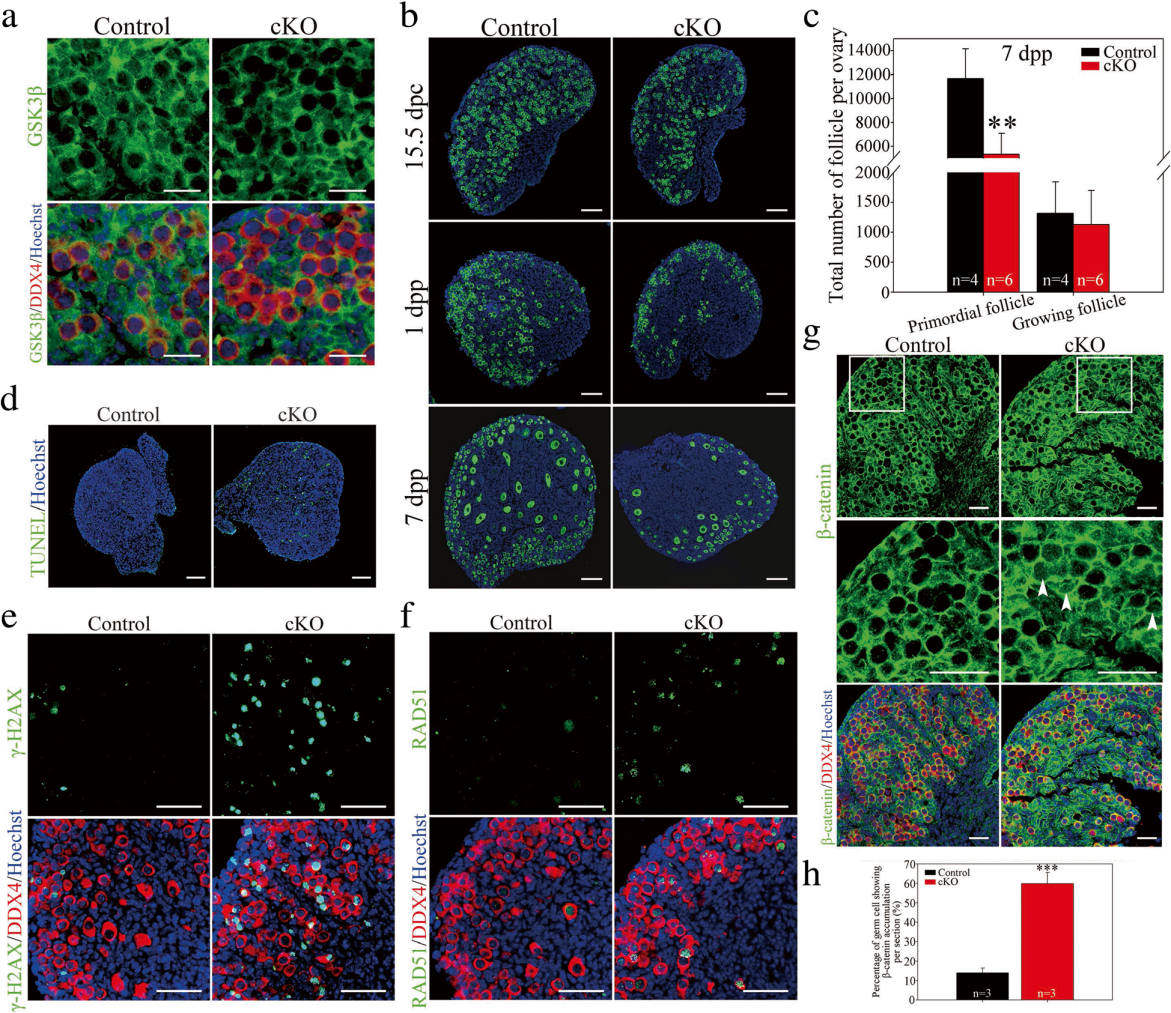
Oocyte-specific deletion of *Gsk-3β* disrupted early folliculogenesis in mice. **a** *Gsk-3β* was efficiently and specifically deleted in the oocytes in cKO mouse ovary. Immunofluorescence staining for GSK-3β (green) and DDX4 (red) on 1 dpp ovary. The nucleus was stained by Hoechst (blue). **b** Control and cKO ovaries at the indicated developmental stages. Oocytes were stained with DDX4 (green). The nucleus was stained by Hoechst (blue). **c** Statistical analysis showed that the total number of primordial follicle decreased significantly in 7 dpp cKO ovaries (Additional file 8: Individual data values). **d** Apoptotic cells increased in 1 dpp cKO ovaries compared with the control ovaries. TUNEL signals (green) marked apoptotic cells. The nucleus was stained by Hoechst (blue). **e** DSBs persisted in the oocytes of 1 dpp cKO ovaries. The sections were stained with γ-H2AX (green) and DDX4 (red). The nucleus was stained by Hoechst (blue). **f** Ectopic RAD51 expression in the oocytes of 1 dpp cKO ovaries. The sections were stained with RAD51 (green) and DDX4 (red). The nucleus was stained by Hoechst (blue). **g**, **h** β-catenin accumulated in the cytoplasm and translocated into the nucleus of the oocytes in cKO mice. **g** The sections were stained with β-catenin (green) and DDX4 (red). The nucleus was stained by Hoechst (blue). Arrowheads indicated oocytes showing nuclear β-catenin accumulation. **h** The statistic analysis demonstrated that the percentage of oocytes showing β-catenin accumulation per section increased significantly in cKO mice (Additional file 8: Individual data values). The data are presented as mean ± s.d. The asterisk (*) denotes a statistically significant difference between the control and treatment groups. *P < 0.05, **P < 0.01, and ***P < 0.001 (*t* test). Scale bars, 200 μm

To identify the physiological role of GSK-3β in developing fetal ovaries in vivo, a germ cell-specific deletion of *Gsk-3β* mouse model was produced. In brief, by crossing *Gsk-3β*^*flox/flox*^ mice with *Ddx4-Cre* mice, *Gsk-3β*^*flox/−*^;*Ddx4-Cre* mice (which exhibited Cre-mediated recombination confined to the germline cells beginning at approximately 15.5 dpc [40]) were produced and are referred to as *Gsk-3β* cKO mice. The wild-type littermates were generally used as the control. Immunofluorescence results verified a significant decrease in GSK-3β expression in the cytoplasm of oocytes in cKO mice compared with that in the control. The conditional deletion strategy did not affect GSK-3β expression in somatic cells, which proved the efficiency and accuracy of Cre-mediated deletion (Fig. 8a).

Next, the effect of germ cell-specific GSK-3β deletion on oogenesis and early folliculogenesis in mice was assessed. Histological sections and immunofluorescence revealed insignificant differences between *Gsk-3β* cKO and the control ovary at 15.5 dpc, while on 1 dpp, when germ cell cyst breakdown and primordial follicle formation was progressing, fewer oocytes and primordial follicles were available in the *Gsk-3β* cKO ovaries compared with those in the control (Fig. 8b). On 7 dpp, the *Gsk-3β* cKO ovary showed a significantly lower follicle reserve than that in the control, and the statistical analysis proved that the number of primordial follicles in the cKO mice was significantly less than that in the control (5338.33 ± 1727.99 in the cKO ovary versus 11,662.50 ± 2459.24 in the control per ovary; *P* < 0.01). However, the numbers of growing follicles in the ovaries showed insignificant differences between the cKO and control ovaries (1130.00 ± 568.11 for the cKO ovary versus 1315.00 ± 528.50 for the control per ovary; *P* > 0.05) (Fig. 8c). To clarify whether fetal oocyte loss following GSK-3β deletion was due to increased apoptosis, the TUNEL (TdT-mediated dUTP Nick-End Labeling) assay was performed on 1 dpp ovaries, which showed that the TUNEL-positive cells in *Gsk-3β* cKO ovaries were obviously more than that in the control, indicating excessive cell apoptosis in the ovaries of *Gsk-3β* cKO mice (Fig. 8d). These observations demonstrate that GSK-3β is essential for fetal oocyte survival during meiotic prophase I in the fetal mouse ovary.

Moreover, the meiotic DSB repair following germ cell-specific *Gsk-3β* deletion was assessed. When 1 dpp ovaries were stained with γ-H2AX, more γ-H2AX-positive oocytes were observed in *Gsk-3β* cKO ovaries compared with those in the control, which further confirmed the sustained DSBs in the oocytes after *Gsk-3β* deletion (Fig. 8e). Similarly, more RAD51 signal-positive oocytes were found in the *Gsk-3β* cKO ovaries than those in the control, which demonstrated incomplete DSB repair after *Gsk-3β* deletion (Fig. 8f). Additionally, we examined the expression of β-catenin in *Gsk-3β-*deleted ovaries. According to the histological section and immunofluorescence results, cytoplasmic accumulation and nuclear translocation of β-catenin in the oocytes of cKO ovaries were detected (arrowhead) (Fig. 8g). Statistical analysis demonstrated that the percentage of oocytes showing β-catenin accumulation per section increased significantly in cKO ovaries (*P* < 0.001) (Fig. 8h). In summary, the results from the *Gsk-3β* cKO mice were in accordance with the in vitro GSK-3β inhibition findings, which indicated that GSK-3β is a prerequisite for fetal oocyte survival in mice.

## Discussion

The results from this study revealed the importance of GSK-3β in protecting oocytes from premature loss in fetal ovaries in mice. Physiologically, the decreased activity of GSK-3β in perinatal oocytes enabled the nuclear translocation of β-catenin, which directly binds to the promoter region of *TAp63* and promotes its transcriptional expression. In vitro or in vivo impaired function of GSK-3β before prophase arrest leads to aberrantly premature expression of TAp63 in fetal oocytes, which induces significant meiotic prophase I oocyte apoptosis in ovaries. These results implied that GSK-3β plays an important role in sustaining fetal oocyte survival before programmed DSB repair completion by fine-tuning the expression of TAp63 in oocytes via restricting the transcriptional activity of β-catenin.

The decreased activity of GSK-3β within oocytes was correlated with the increased expression level of TAp63 in fetal and neonatal oocytes in the physiological condition. The protein level of GSK-3β in oocytes was relatively consistent from 13.5 dpc to 1 dpp; however, the inactive mode, p-GSK-3β (Ser9), was evidently upregulated after 17.5 dpc, which indicated the downregulation of GSK-3β activity within oocytes. Coincidentally, the mRNA level of *TAp63* started to increase significantly from 17.5 dpc onward. The protein expression of TAp63 was detectable in approximately 20% of the oocytes in 18.5 dpc ovaries according to previous reports and our results [18], and the expression peak of TAp63 appeared on 1 dpp, when the massive fetal oocyte attrition occurred in vivo, which implied that TAp63 exerted function in sensing defects and eliminating defective oocytes. From 5 dpp onward, the expression of TAp63 remained high in the oocytes of primordial and primary follicles, implying a long-term guardian role of TAp63 in the adult ovary [18]. Therefore, we assumed that the mutual spatiotemporal expression patterns of GSK-3β and TAp63 might be closely related to the PCD of oocytes in the perinatal mouse ovary.

Although fetal oocytes in the early stages of meiotic prophase I tolerate hundreds of DSBs as part of the inherent process of homologous recombination, postnatal oocytes with less than ~ 10 DSBs generally trigger PCD program [13, 41]. Oocytes in follicles employ DNA damage response signaling to supervise DSB generation and to cull the faulty gametes. Generally, ATM senses endogenous DSBs and propagates signaling to CHK2, which blocks the cell cycle, promotes DSB repair, and, in turn, signals to both p53 and p63 in the oocytes [42, 43]. In response to chemotherapy, the activity of TAp63 has been shown to be modulated by CHK2 and casein kinase 1 (CK1) actions, which orchestrate the induction of apoptosis in oocytes [41, 44]. However, the underlying mechanism that coordinates the substantially programmed meiotic DSB existence and the DNA damage checkpoint in fetal oocytes is still elusive. In agreement with other studies, we found that fetal oocytes lack TAp63 surveillance before the diplotene stage, despite the presence of a normal response to the DSB-induced DNA damage checkpoint signaling (ATM and CHK2) [15, 16, 45]. Coincidentally, the expression peak of TAp63 in oocytes was synchronized with the physiologically massive oocyte attrition in ovaries perinatally, with the assumed goal of eliminating oocytes with unrepaired DSBs or genetic defects [13]. In this study, inhibition of GSK-3β resulted in the premature upregulation of TAp63 via β-catenin transcriptional activation, simultaneously leading to oocyte loss in fetal ovaries. However, GSK-3β inhibition failed to trigger significant oocyte attrition in perinatal ovaries. Thus, fetal oocyte attrition following GSK-3β inhibition possibly resulted from the premature TAp63 expression within the oocytes when a significant amount of DSB repair had not yet been completed. It is therefore assumed that the synchronized intensive TAp63 expression and unrepaired DSBs on chromosomes before the pachytene stage are detrimental to fetal oocyte survival, as well as to in vivo primordial follicle formation. GSK-3β might function to coordinate the faithful meiotic progression and timely expression of TAp63 for the sake of oocyte maintenance in fetal ovaries.

Upon the disrupted DNA damage checkpoint expression in ovaries following GSK-3β inhibition, massive oocyte attrition occurred through the apoptosis pathway. Based on the observations from immunofluorescence co-staining studies, we detected increased expression of apoptotic signaling within oocytes in GSK-3β-inhibited ovaries, which provided additional evidence that fetal oocyte population could be diminished through the apoptosis pathway [11]. Intriguingly, those apoptotic oocytes were labeled majorly in the medulla part of the ovaries, which implied that GSK-3β was indispensable for the survival of oocytes from the medulla of ovaries; however, the underlying mechanism that determined the spatial distinction required further investigations. Moreover, since each primordial follicle is composed of single oocyte and several pre-granulosa cells, the number of oocyte reserve may exert influence on folliculogenesis in ovaries. According to our findings, following GSK-3β inhibition, the number of oocytes reduced significantly during 14.5 dpc + 4 days but maintained almost unchanged during 17.5 dpc + 4 days. Whereas, the number of formed primordial follicle decreased sharply in ovaries treated with GSK-3β inhibitor for 14.5 dpc + 7 days. These results implied that the reduced primordial follicle formation might result from the reduced number of oocytes during 14.5 to 18.5 dpc. Thus, we assumed that blockage of GSK-3β not only impacted oocyte survival during meiotic prophase I, but also causally impaired subsequent folliculogenesis in mouse ovaries.

In addition, we found that inhibition of GSK-3β caused meiotic progression delay and meiotic DSB repair deficiency in fetal oocytes in mice. As has been stated, programmed DSB formation and repair were induced in oocytes during meiotic prophase I to enable recombination. Intriguingly, TAp63 activity was blocked during meiotic DSB repair, which might be a protective restraining mechanism to avoid disordered germ cell loss during meiotic prophase I [45]. Nevertheless, according to our results, TAp63 was prematurely expressed in oocytes following GSK-3β inhibition as early as 16.5 dpc in mice, given that oocytes from both the control and the treated ovaries were tolerating massive meiotic DSBs around 16.5 dpc; while the expression of TAp63 was detected in GSK-3β-inhibited oocytes, we assumed that the aberrant expression of TAp63 might result from GSK-3β inhibition, instead of persisting unrepaired DSBs triggered TAp63 expression. According to the existed findings, oocytes generally triggered DNA damage response, including cell cycle arrest or apoptotic cell death once the DNA damage checkpoint signaling was activated [43, 46]. In line with this, we observed disturbed meiotic progression and continuous germ cell loss following GSK-3β inhibition. Importantly, when the aberrant expression of TAp63 was prevented via antagonizing the transcriptional activity of β-catenin, the DSB repair deficiency could be reduced efficiently. Hence, it is speculated that GSK-3β is correlated to faithful meiotic progression via time-dependently regulating the appearance of TAp63 during meiotic prophase I. However, substantial studies are still needed to address the detailed regulatory role of TAp63 in meiotic progression and DSB repair in the future.

As the core mediator in the canonical WNT/β-catenin signaling pathway, β-catenin has been revealed to be vital to gonad development [47, 48]. While active β-catenin signaling in somatic cells is necessary for maintaining female gonad development [49, 50], overactivation of β-catenin signaling in germline cells may be destructive for gametogenesis. Stabilization and nuclear localization of β-catenin in PGCs caused germ cell deficiency with delayed cell cycle progression [51]. Therefore, it is proposed that the cell type-dependent and developmental stage-specific interaction of the WNT/β-catenin pathway is determinative for female fertility. The present results showed that β-catenin displayed a spatiotemporal cytoplasmic-nuclear translocation in fetal oocytes, which acted on the transcriptional activation of *TAp63* expression in vivo. Meanwhile, following GSK-3β ablation, aberrant nuclear translocation of β-catenin is responsible for fetal oocyte attrition, as antagonism of the transcriptional activity of β-catenin rescued oocytes from apoptosis in the fetal ovary. Consistent with previous reports, we demonstrated that a delicately balanced β-catenin signaling, which is negatively controlled by GSK-3β activity in the fetal ovary, is pivotal for early oogenesis in mice.

In this study, although germ cell-conditional deletion of *Gsk-3β* resulted in decreased ovarian reserve, the number of growing follicle was insignificantly different between the cKO and the control mice in 7 dpp ovaries. Considering that the oocytes initiated meiosis asynchronously from 13.5 dpc, yet the expression of *Ddx4*-Cre recombinase started from 15 to 18 dpc in germ cells [40], it is assumed that the equivalent amount of growing follicle in the cKO and the control mouse ovary might be arised from the oocytes which entered meiosis and had reached the pachytene stage prior to *Gsk-3β* deletion.

## Conclusions

In conclusion, GSK-3β plays an indispensable role in protecting fetal oocytes from apoptosis during meiotic prophase I via impeding the transcriptional activation of *TAp63* expression in fetal oocytes. In the physiological condition, with the decreasing GSK-3β activity perinatally, the nuclear translocation of β-catenin results in the upregulation of *TAp63* expression, which is targeted at initiating PCD in defective oocytes. However, in vitro or in vivo blockage of GSK-3β before the programmed DSB repair completion results in premature expression of TAp63 in fetal oocytes, which mistakenly triggers a cull of fetal oocytes (Fig. 7d). This study uncovered a novel molecular regulatory mechanism of the GSK-3β protective function and TAp63-induced PCD in fetal oocyte dynamics before the meiotic arrest, which may provide insight into the pathogenic mechanism of POI or reproductive disease caused by a genetic deficiency in fetal oocytes.

## Materials and methods

### Animals

All wild-type mice were purchased from the Laboratory Animal Center of the Institute of Genetics (Beijing, China). The *Gsk-3β*^*flox/flox*^ mice were generally provided by Dr. J. Woodgett (Mount Sinai Hospital, Canada). The *Ddx4-Cre* mice were obtained from Professor Hua Zhang (College of Biological Sciences, China Agricultural University, China). *Gsk-3β*^*flox/flox*^ mice were crossed with *Ddx4-Cre* mice to produce *Gsk-3β*^*flox*/*−*^*;Ddx4-Cre* mice as germ cell-conditional KO (cKO), and wild-type littermates were used as the control. Genotyping was performed by PCR using mouse tail genomic DNA, and primer sets were displayed in Additional file 5: Table S1. All mice were housed with 16/8 h light/dark cycles, at 26 °C and allowed free access to water and food. Female mice were mated with males overnight, and the following day was designated as 0.5 dpc when the presence of a vaginal plug was confirmed. All experiments were performed in accordance with institutional and national guidelines and regulations and were approved by the China Agricultural University Animal Care and Use Committee.

### Ovary isolation and in vitro culture

Ovaries were dissected carefully from mice on the designated time in pre-chilled phosphate-buffered saline (PBS) (10 mM, pH = 7.4) under a stereomicroscope (ZSA302, COIC, China) in sterile conditions. The isolated ovaries were transferred to and immersed in 1 ml of culture media in individual wells of a 6-well plate (NEST Biotechnology, Wuxi, Jiangsu, China) and incubated in a 37 °C incubator with 5% CO_2_. Serum-free Dulbecco’s modified Eagle’s medium/Ham’s F12 nutrient mixture (DMEM/F12, Gibco BRL, Carlsbad, CA, USA) supplemented with 10 IU/ml penicillin-streptomycin and HEPES was used for in vitro fetal ovary culture. BIO (B1686; Sigma Chemical Co., St. Louis, MO, USA), CHIR99021 (S1263; Selleck, Shanghai, China), and ICG-001 (S2662; Selleck, Shanghai, China) were dissolved in DMSO. The final concentrations of BIO, CHIR99021, and ICG-001 were 1 μM, 5 μM and 0.5 μM, respectively. The control group was supplemented with the same volume of DMSO.

### Chromatin immunoprecipitation

The ChIP assays were performed using a MAGNA ChIP kit (Millipore) according to the manufacturer’s protocol. 17.5 dpc ovaries were collected, digested, and sheared by sonication until the average DNA length was about 300–500 bp, as evaluated by 2% agarose gel electrophoresis. Chromatin fragments were incubated with 3 μg anti-β-catenin antibodies (AC106, Beyotime Biotechnology, Shanghai, China) or normal rabbit IgG (Santa Cruz Biotechnology) overnight at 4 °C. Enriched DNA was quantified by real-time PCR. Primers are listed in Additional file 6: Table S2.

### Histological sections and immunofluorescence

Collected ovaries were fixed in 4% paraformaldehyde (PFA) in PBS overnight, then transferred to 70% ethyl alcohol, dehydrated and embedded in paraffin, and cut into 5-μm-thick sections. The sections were deparaffinized, rehydrated, and subjected to antigen retrieval using 0.01% sodium citrate buffer (pH = 6.0). Then, sections were blocked with 10% normal donkey serum in PBS for 1 h at room temperature and incubated with primary antibodies overnight at 4 °C. Primary antibody dilution: GSK-3β (1:100; #12456P, Cell Signaling Technology, MA, USA), DDX4 (1:200; #ab27591, Abcam, MA, USA), cleaved Caspase-3 (1:100; #AC033, Beyotime Biotechnology, Shanghai, China), MSY2 (1:200; #sc-21316, Santa Cruz Biotechnology, CA, USA), PCNA (1:100; #sc-56, Santa Cruz Biotechnology, CA, USA), BrdU (1:100; #G3G4, DSHB, USA), SYCP3 (1:200; #sc-20845, Santa Cruz Biotechnology, CA, USA), β-catenin (1:200; #sc-7199, Santa Cruz Biotechnology, CA, USA), active β-catenin (1:100; #05-665, Millipore, USA), p-ATM (1:100; #AA866-1, Beyotime Biotechnology, Shanghai, China), p-CHK2 (1:100; #BS4043, Bioworld, MO, USA), p63 (1:100; #BS1279, Bioworld, MO, USA), RAD51 (1:100; #sc-8349, Santa Cruz Biotechnology, CA, USA), and γ-H2AX (1:200; #NB100-2280, NOVUS, CO, USA) (Additional file 7: Table S3). Next, sections were rinsed thoroughly in PBS and incubated with fluorophore-conjugated secondary antibody dissolved in PBS for 1 h at 37 °C. Second antibody dilution: Alexa Fluor 488- or 555-conjugated donkey secondary antibodies against mouse/rabbit IgG (1:200, Life Technologies, USA). The sections were then rinsed in PBS, stained with Hoechst 33342 (B2261, Sigma, USA) for 5 min, and sealed in anti-fade fluorescence mounting medium (Applygen, China) with coverslips. The sections were examined and photographed using Nikon Eclipse 80i digital fluorescence microscope or Nikon A1 laser scanning confocal microscope.

### Oocytes and primordial follicle quantification

The 5-μm serial sections were stained with an antibody against DDX4 and Hoechst, and the numbers of oocytes and follicle were counted in every fifth section. The sum was multiplied by five to estimate the total numbers of oocytes and follicle per ovary. The primordial follicles were distinguished as a single oocyte surrounded by several flattened pre-granulose cells.

### Germ cell proliferation analysis

The percentage of germ cells in S-phase was evaluated by measuring the BrdU incorporation using the Cell Proliferation Kit (GE Healthcare, Buckinghamshire, UK) according to the manufacturer’s instructions. In brief, BrdU (1%) was added into the culture media 1 h before collection. Paraffin sections and immunofluorescence were performed as described above. The analysis was performed by counting the number of cells co-staining for both BrdU and DDX4 antibody per section.

### TUNEL assay

Paraffin sections were prepared as described above. Apoptosis was evaluated by TUNEL staining using the Click-iT Plus TUNEL Assay (C10617; Life Technologies, USA), according to the manufacturer’s protocol. To determine the apoptotic oocytes, the number of TUNEL-positive cells with larger size and spherical nucleus was recorded in each section.

### Meiotic spread preparations and immunofluorescence staining

Briefly, ovaries were digested with 1% trypsin and dispersed ovarian cells were collected in 1% PFA containing 2% Triton X-100, then dripped on a slide and fixed for 6 h at room temperature. The slides were blocked with ADB (antibody dilution buffer; containing 1% normal donkey serum, 3‰ BSA, and 1‰ Triton X-100 in TBS) for 30 min at room temperature and incubated with the appropriate primary antibodies overnight at 37 °C. Primary antibody dilution: SYCP3 (1:50; #sc-20845, Santa Cruz Biotechnology, CA, USA), RAD51 (1:50; #sc-8349, Santa Cruz Biotechnology, CA, USA), and γ-H2AX (1:100; #NB100-2280, NOVUS, CO, USA) (Additional file 7: Table S3). The slides were rinsed thoroughly in PBS and incubated with second antibodies (1:200; Life Technologies, USA) dissolved in PBS for 1 h at 37 °C. The sections were then rinsed in PBS, stained with Hoechst 33342 (B2261, Sigma, USA) for 5 min, and sealed in anti-fade fluorescence mounting medium (Applygen, China) with coverslips. The sections were examined and photographed using Nikon Eclipse 80i digital fluorescence microscope. The average percentages of germ cells staining with designated antibody were determined from three slides, each of which included at least three ovaries. Germ cells were randomly dispersed on the slides, and at least 300 germ cells were analyzed per slide. Unidentified germ cells were not included in the analysis.

### RNA extraction and real-time qRT-PCR

Total RNA from ten ovaries was extracted using TRIzol reagent (Invitrogen, Carlsbad, CA, USA), according to the manufacturer’s protocol; washed in 75% ethanol; and then dissolved in water. First-strand cDNA was synthesized by reverse transcription (Promega Reverse Transcription System) from 1 μg of total RNA. The PCR was performed on an ABI 7500 Sequence Detection System (Applied Biosystems) using the following parameters: 10 min at 95 °C, 40 cycles of 15 s at 95 °C, and 1 min at 60 °C. Gene expression changes were evaluated using real-time qRT-PCR in 15 μl reaction volumes and normalized to *β-actin*.

### Western blotting analysis

Total protein from ten ovaries was extracted in WIP Tissue and Cell lysis solution containing 1 mM phenylmethylsulfonyl fluoride (Cell Signaling Technologies, USA) according to the manufacturer’s instructions. The samples were separated on 10% SDS-PAGE and then transferred to polyvinylidene fluoride (PVDF) membranes (IPVH00010, Millipore, USA), which were incubated with appropriate primary antibodies overnight at 4 °C. Primary antibody dilution: GSK-3β (1:200; #12456P, Cell Signaling Technology, MA, USA), p-GSK-3β (1:200; #AG753-1, Beyotime Biotechnology, Shanghai, China), β-catenin (1:500; #sc-7199, Santa Cruz Biotechnology, CA, USA), p-β-catenin (1:500; #9561S, Cell Signaling Technology, MA, USA), p63 (1:200; # BS1279, Bioworld, MO, USA), DDX4 (1:1000; #ab27591, Abcam, MA, USA), FOXL2 (1:1000; #ab5096, Abcam, MA, USA), Caspase-3 (1:1000; #ab32351, Abcam, MA, USA), and GAPDH (1:1000; #AM4300, Ambion, USA) (Additional file 7: Table S3). GAPDH was used as an internal control. The membranes were rinsed thoroughly with TBST and incubated with peroxidase-conjugated secondary antibody (1:5000; #ZB2301, ZSGB-BIO, China) at room temperature for 1 h. The membranes were then rinsed thoroughly with TBST and visualized using SuperSignal West Pico chemiluminescent detection system (Prod 34080, Thermo, USA).

### Preparation of disassociated ovarian cells

Ovaries from prescribed dpc were collected respectively as described previously. After incubated in 100 μl trypsin solution at 37 °C for 5–10 min, tissues were pipetted up and down for digestion. When the tissues were digested into single-cell suspensions, the digestion reaction was terminated by 20% fetal bovine serum (FBS). The cell suspension was centrifuged at 4 °C, 1000*g* for 5 min to collect precipitation, then re-suspended in 1 ml PBS and washed thoroughly. The cell suspension was then centrifuged at 4 °C, 1000*g*, and collected in DMEM/F12 with 4% FBS and 1% modified iTS (Insulin-Transferrin-Selenium Solution; 51500056, Life Technology, USA) supplement. The ovarian cells suspended in DMEM/F12/iTS were cultured in 24-well plates at 37 °C, 5% CO_2_ for 6–8 h. Plates were gently shaken to flush out the loosely adhered oocytes, and supernatants were collected for centrifugation to collect the oocyte component. The somatic cell component that adhered to the culture plate was recovered by digestion using 0.25% trypsin. Collected cells were cleaned in PBS for further examination.

### Statistical analysis

The data were reported as the mean ± s.d. of results performed in triplicate. Three ovaries per group were used for the meiotic spreading and the oocytes and primordial follicle quantification experiment. The quantification results and real-time qRT-PCR data were analyzed by *t* tests or analysis of variance (ANOVA) using Sigmaplot version 9.01 software. Differences were considered significant at *P* < 0.05.

## Additional files

Additional file 1: **Figure S1.** Expression of GSK-3β in the oocyte component of mouse ovaries. (A) Western blotting analysis of the oocyte component from mouse ovaries with germ cell marker (DDX4) and somatic cell marker forkhead box L2 (FOXL2). GAPDH was used as an internal control. The separated oocyte component contained scarce FOXL2 protein, which indicated the oocyte sample was relatively pure. (B) The expression level of GSK-3β in oocyte component from 13.5 dpc to 1 dpp. Western blotting analysis showed that the total GSK-3β was expressed consistently in fetal and neonatal oocytes, while p-GSK-3β displayed increased expression level. GAPDH was used as an internal control. (PDF 795 kb)

Additional file 2: **Figure S2.** Inhibition of GSK-3β did not affect PGC proliferation and meiosis initiation. (A)(B)(C)(D) PGC proliferation was not affected in fetal ovary following GSK-3β inhibition. Before the examination, ovaries at 12.5 dpc were cultured in vitro with DMSO or BIO for 2 days. (A) Sections were stained with BrdU (green) and DDX4 (red). The nucleus was stained by Hoechst (blue). (B) Statistical analysis showed that the proliferating PGCs (co-staining for both BrdU and DDX4) per section displayed an insignificant difference between the control and treatment groups (Additional file 8: individual data values). (C) Sections were stained with PCNA (green) and DDX4 (red). The nucleus was stained by Hoechst (blue). (D) Statistical analysis showed that the number of PCNA-positive oocytes per section displayed an insignificant difference between the control and treatment groups (Additional file 8: Individual data values). (E) Meiosis initiation was not affected in fetal ovary following GSK-3β inhibition. Before the examination, ovaries at 12.5 dpc were cultured in vitro with DMSO or BIO for 2 days. Sections were stained with SYCP3 (green) and DDX4 (red). The nucleus was stained by Hoechst (blue). Ovaries at 13.5 dpc were used as a negative control, as oocytes are devoid of SYCP3 signal in nuclei before meiosis initiation. The majority of oocytes from both the control and treatment group entered meiotic prophase. The data are presented as mean ± s.d. The asterisk (*) denotes a statistically significant difference between the control and treatment groups. *P < 0.05, **P < 0.01, and ***P < 0.001 (*t* test). Scale bars, 200 μm. (PDF 1878 kb)

Additional file 3: **Figure S3.** Inhibition of GSK-3β led to fetal oocyte loss but did not affect germ cyst breakdown and primordial follicle formation perinatally. (A)(B) Inhibition of GSK-3β with CHIR99021 led to dramatic oocyte loss. Before the examination, ovaries at 14.5 dpc were cultured in vitro with DMSO or CHIR99021 for 4 days. (A) Oocytes were stained with DDX4 (green). The nucleus was stained by Hoechst (blue). (B) Statistical analysis showed that the total number of oocytes decreased significantly following CHIR99021 treatment for 4 days (Additional file 8: Individual data values). (C) Inhibition of GSK-3β caused severe apoptosis in fetal ovaries. Before the examination, ovaries at 14.5 dpc were cultured in vitro with DMSO or BIO for 3 days. Western blotting analysis showed the increased Caspase-3 level in fetal ovaries following GSK-3β inhibition. GAPDH was used as an internal control. (D)(E) Inhibition of GSK-3β did not impair germ cell cyst breakdown and primordial follicle formation perinatally. Before the examination, ovaries at 17.5 dpc were cultured in vitro with DMSO or BIO for 4 days. (D) Germ cells were stained with DDX4 (green). The nucleus was stained by Hoechst (blue). Primordial follicle (arrowhead) assemble was intact. (E) Statistical analysis showed that the total number of germ cell and formed primordial follicle displayed an insignificant difference between the control and treatment groups (Additional file 8: Individual data values). (F)(G) Inhibition of GSK-3β impaired folliculogenesis. Before the examination, ovaries at 14.5 dpc were cultured in vitro with DMSO or BIO for 7 days. (F) Germ cells were stained with DDX4 (green). The nucleus was stained by Hoechst (blue). (G) Statistical analysis showed that the total number of follicle displayed a significant difference between the control and treatment groups (Additional file 8: Individual data values). The data are presented as mean ± s.d. The asterisk (*) denotes a statistically significant difference between the control and treatment groups. *P < 0.05, **P < 0.01, and ***P < 0.001 (*t* test). Scale bars, 200 μm. (PDF 1475 kb)

Additional file 4: **Figure S4.** The expression pattern of DNA damage checkpoint signaling in fetal and neonatal mouse ovary in vivo. (A). Percentage of meiotic substage in 15.5 dpc, 17.5 dpc, and 1 dpp ovaries in vivo (bar chart). Percentage of γ-H2AX-positive germ cells in 15.5 dpc, 17.5 dpc, and 1 dpp ovaries in vivo (line chart) (Additional file 8: Individual data values). (B). Mouse ovaries from 13.5 dpc, 15.5 dpc, 17.5 dpc, and 1 dpp were immunostained for p-ATM (green) and DDX4 (red). The nucleus was stained by Hoechst (blue). p-ATM displayed intensive expression in the oocyte nucleus from 15.5 to 17.5 dpc. (C). Mouse ovaries from 13.5 dpc, 15.5 dpc, 17.5 dpc, and 1 dpp were immunostained for p-CHK2 (green) and DDX4 (red). The nucleus was stained by Hoechst (blue). p-CHK2 displayed intensive expression in the oocyte nucleus from 15.5 to 17.5 dpc. (D). qRT-PCR analysis of mRNA expression level of *TAp63* in mouse ovaries from 13.5 dpc to 1 dpp (normalized to *β-actin*). *TAp63* expression level displayed significant increase from 17.5 dpc onward (Additional file 8: Individual data values). The data are presented as mean ± s.d. Different letters (a–c) denote a statistically significant difference between groups (ANOVA and Holm-Sidak test). Scale bars, 200 μm. (PDF 1549 kb)

Additional file 5: **Table S1.** Genotyping primers. (DOCX 11 kb)

Additional file 6: **Table S2.** ChIP-qPCR primers. (DOCX 11 kb)

Additional file 7: **Table S3.** Antibody information. (DOCX 13 kb)

Additional file 8: Individual data values. (XLSX 20 kb)
