# Supplementary figures and images for: GSK-3β protects fetal oocytes from premature death via modulating TAp63 expression in mice
Source: BMC Biol. 2019 Mar 12;17:23. doi: 10.1186/s12915-019-0641-9 (PMC6417224; doi:10.1186/s12915-019-0641-9)

Figure S1

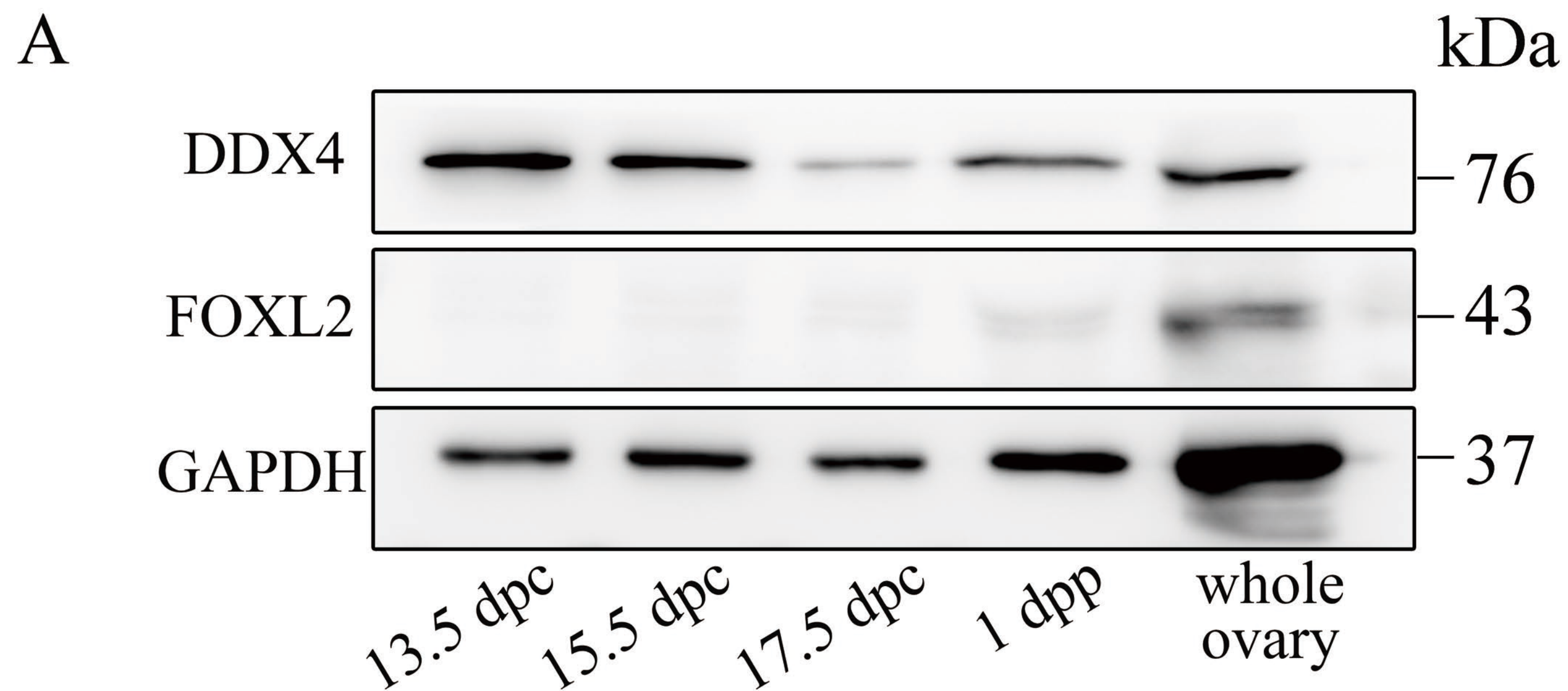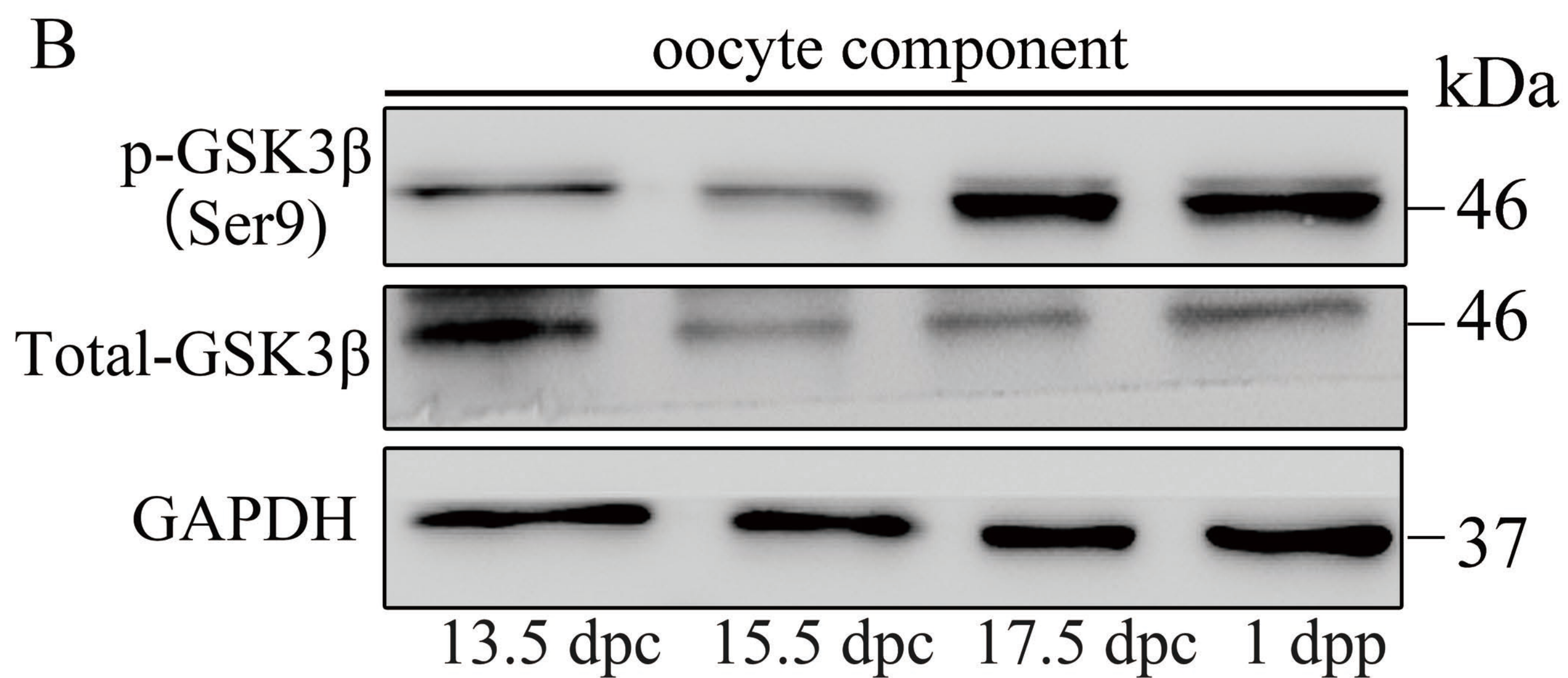

Supplement: Supplementary file 1 — Figure S1. Expression of GSK-3β in the oocyte component of mouse ovaries. (A) Western blotting analysis of the oocyte component from mouse ovaries with germ cell marker (DDX4) and somatic cell marker forkhead box L2 (FOXL2). GAPDH was used as an internal control. The separated oocyte component contained scarce FOXL2 protein, which indicated the oocyte sample was relatively pure. (B) The expression level of GSK-3β in oocyte component from 13.5 dpc to 1 dpp. Western blotting analysis showed that the total GSK-3β was expressed consistently in fetal and neonatal oocytes, while p-GSK-3β displayed increased expression level. GAPDH was used as an internal control. (PDF 795 kb) [file 12915_2019_641_MOESM1_ESM.pdf]

## Figure S2

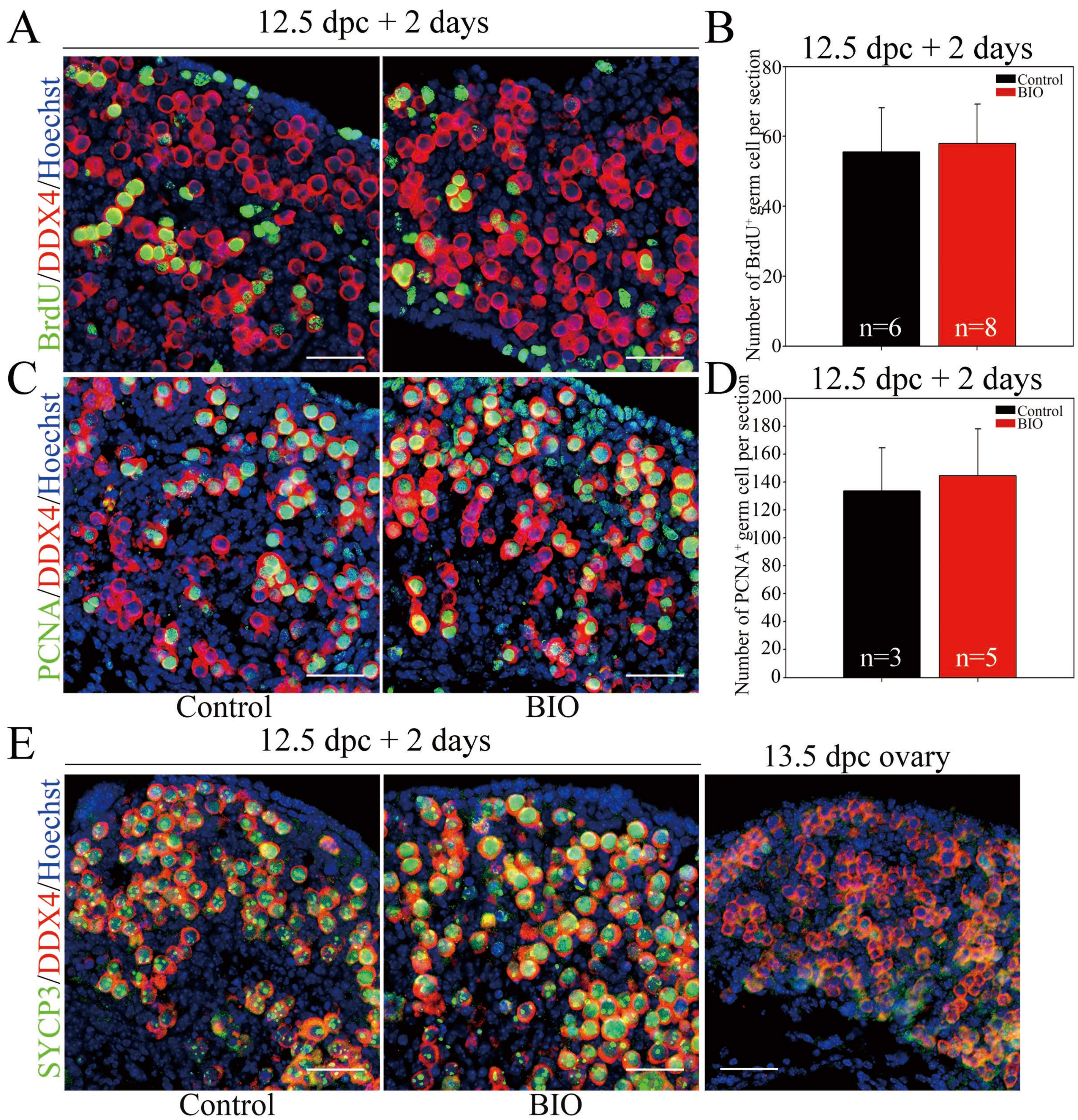

Supplement: Supplementary file 2 — Figure S2. Inhibition of GSK-3β did not affect PGC proliferation and meiosis initiation. (A)(B)(C)(D) PGC proliferation was not affected in fetal ovary following GSK-3β inhibition. Before the examination, ovaries at 12.5 dpc were cultured in vitro with DMSO or BIO for 2 days. (A) Sections were stained with BrdU (green) and DDX4 (red). The nucleus was stained by Hoechst (blue). (B) Statistical analysis showed that the proliferating PGCs (co-staining for both BrdU and DDX4) per section displayed an insignificant difference between the control and treatment groups (Additional file 8: individual data values). (C) Sections were stained with PCNA (green) and DDX4 (red). The nucleus was stained by Hoechst (blue). (D) Statistical analysis showed that the number of PCNA-positive oocytes per section displayed an insignificant difference between the control and treatment groups (Additional file 8: Individual data values). (E) Meiosis initiation was not affected in fetal ovary following GSK-3β inhibition. Before the examination, ovaries at 12.5 dpc were cultured in vitro with DMSO or BIO for 2 days. Sections were stained with SYCP3 (green) and DDX4 (red). The nucleus was stained by Hoechst (blue). Ovaries at 13.5 dpc were used as a negative control, as oocytes are devoid of SYCP3 signal in nuclei before meiosis initiation. The majority of oocytes from both the control and treatment group entered meiotic prophase. The data are presented as mean ± s.d. The asterisk (*) denotes a statistically significant difference between the control and treatment groups. *P < 0.05, **P < 0.01, and ***P < 0.001 (t test). Scale bars, 200 μm. (PDF 1878 kb) [file 12915_2019_641_MOESM2_ESM.pdf]

Figure S3

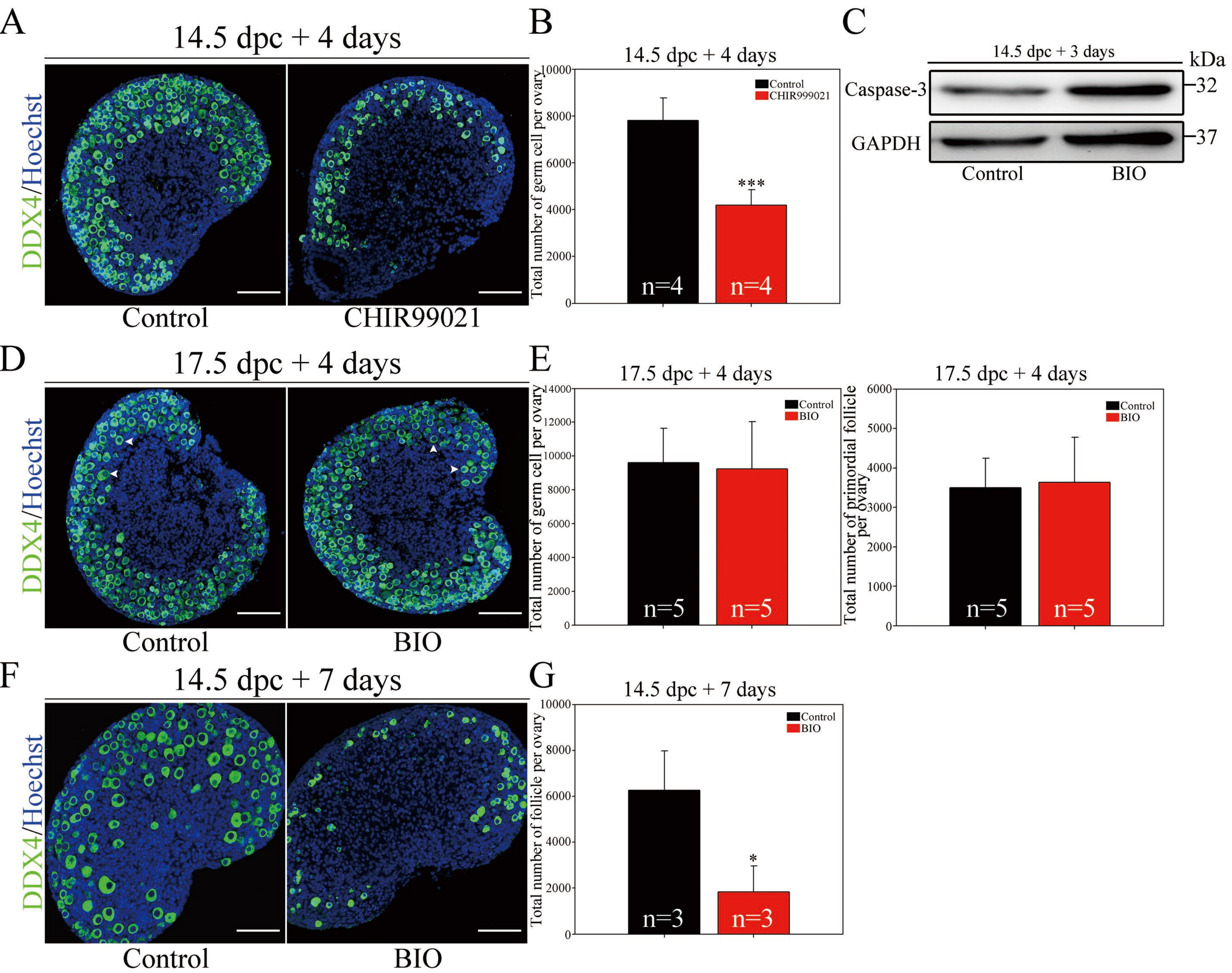

Supplement: Supplementary file 3 — Figure S3. Inhibition of GSK-3β led to fetal oocyte loss but did not affect germ cyst breakdown and primordial follicle formation perinatally. (A)(B) Inhibition of GSK-3β with CHIR99021 led to dramatic oocyte loss. Before the examination, ovaries at 14.5 dpc were cultured in vitro with DMSO or CHIR99021 for 4 days. (A) Oocytes were stained with DDX4 (green). The nucleus was stained by Hoechst (blue). (B) Statistical analysis showed that the total number of oocytes decreased significantly following CHIR99021 treatment for 4 days (Additional file 8: Individual data values). (C) Inhibition of GSK-3β caused severe apoptosis in fetal ovaries. Before the examination, ovaries at 14.5 dpc were cultured in vitro with DMSO or BIO for 3 days. Western blotting analysis showed the increased Caspase-3 level in fetal ovaries following GSK-3β inhibition. GAPDH was used as an internal control. (D)(E) Inhibition of GSK-3β did not impair germ cell cyst breakdown and primordial follicle formation perinatally. Before the examination, ovaries at 17.5 dpc were cultured in vitro with DMSO or BIO for 4 days. (D) Germ cells were stained with DDX4 (green). The nucleus was stained by Hoechst (blue). Primordial follicle (arrowhead) assemble was intact. (E) Statistical analysis showed that the total number of germ cell and formed primordial follicle displayed an insignificant difference between the control and treatment groups (Additional file 8: Individual data values). (F)(G) Inhibition of GSK-3β impaired folliculogenesis. Before the examination, ovaries at 14.5 dpc were cultured in vitro with DMSO or BIO for 7 days. (F) Germ cells were stained with DDX4 (green). The nucleus was stained by Hoechst (blue). (G) Statistical analysis showed that the total number of follicle displayed a significant difference between the control and treatment groups (Additional file 8: Individual data values). The data are presented as mean ± s.d. The asterisk (*) denotes a statistically significant difference betw [file 12915_2019_641_MOESM3_ESM.pdf]

Figure S4

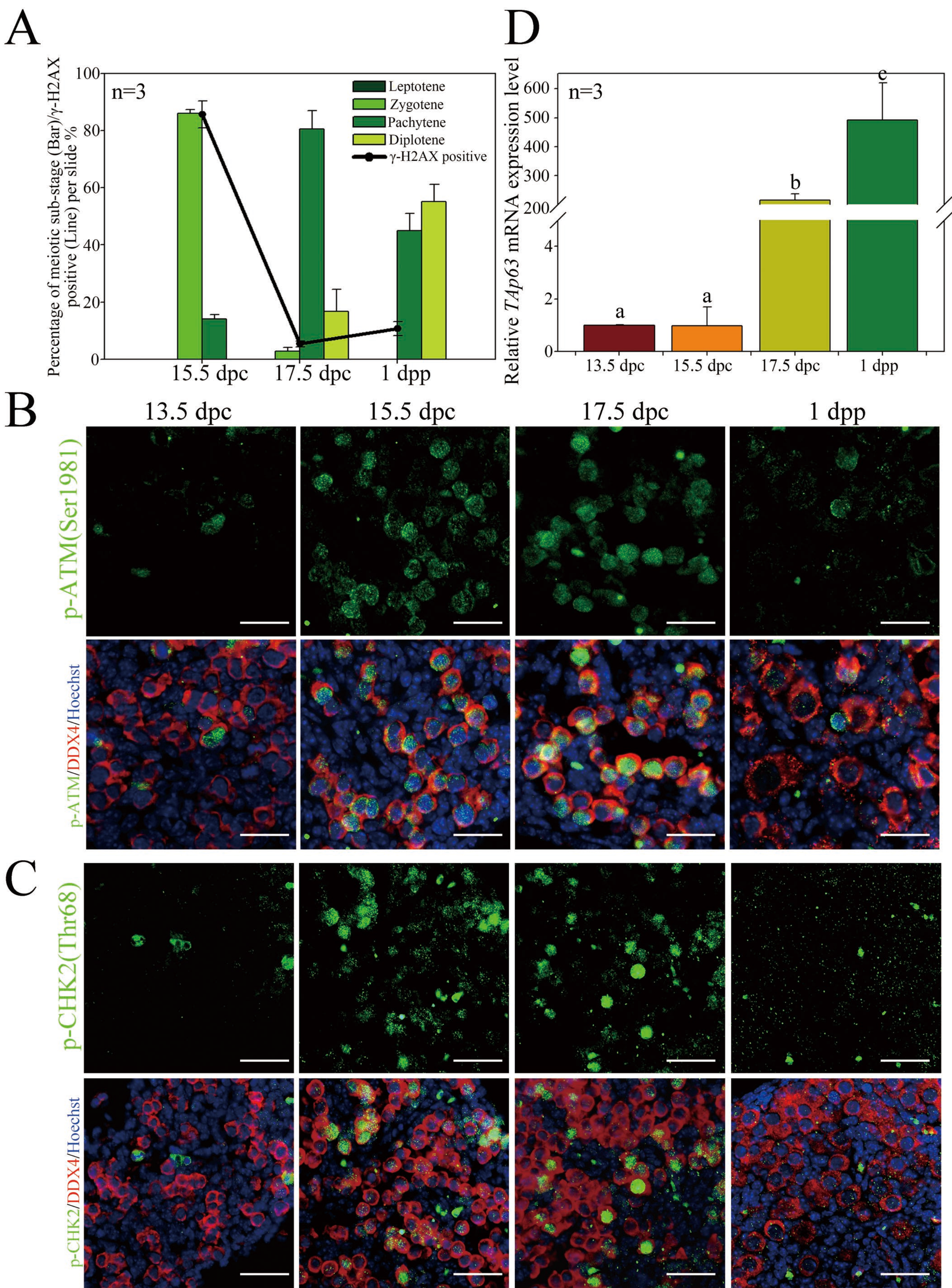

Supplement: Supplementary file 4 — Figure S4. The expression pattern of DNA damage checkpoint signaling in fetal and neonatal mouse ovary in vivo. (A). Percentage of meiotic substage in 15.5 dpc, 17.5 dpc, and 1 dpp ovaries in vivo (bar chart). Percentage of γ-H2AX-positive germ cells in 15.5 dpc, 17.5 dpc, and 1 dpp ovaries in vivo (line chart) (Additional file 8: Individual data values). (B). Mouse ovaries from 13.5 dpc, 15.5 dpc, 17.5 dpc, and 1 dpp were immunostained for p-ATM (green) and DDX4 (red). The nucleus was stained by Hoechst (blue). p-ATM displayed intensive expression in the oocyte nucleus from 15.5 to 17.5 dpc. (C). Mouse ovaries from 13.5 dpc, 15.5 dpc, 17.5 dpc, and 1 dpp were immunostained for p-CHK2 (green) and DDX4 (red). The nucleus was stained by Hoechst (blue). p-CHK2 displayed intensive expression in the oocyte nucleus from 15.5 to 17.5 dpc. (D). qRT-PCR analysis of mRNA expression level of TAp63 in mouse ovaries from 13.5 dpc to 1 dpp (normalized to β-actin). TAp63 expression level displayed significant increase from 17.5 dpc onward (Additional file 8: Individual data values). The data are presented as mean ± s.d. Different letters (a–c) denote a statistically significant difference between groups (ANOVA and Holm-Sidak test). Scale bars, 200 μm. (PDF 1549 kb) [file 12915_2019_641_MOESM4_ESM.pdf]
