# Supplementary material for: GSK-3β protects fetal oocytes from premature death via modulating TAp63 expression in mice
Source: BMC Biol. 2019 Mar 12;17:23. doi: 10.1186/s12915-019-0641-9 (PMC6417224; doi:10.1186/s12915-019-0641-9)
Supplement: Supplementary file 5 — Table S1. Genotyping primers. (DOCX 11 kb) [file 12915_2019_641_MOESM5_ESM.docx]

**Table S1. Genotyping primers.**

| **Gene** | **Forward (5’to 3’)** | **Reverse (5’to 3’)** |
| --- | --- | --- |
| *Gsk-3β* flox | GGGGCAACCTTAATTTCATT | GTGTCTGTATAACTGACTTCCTGTGGC |
| *Gsk-3β* delete | GGGGCAACCTTAATTTCATT | TCTGGGCTATAGCTATCTAGTAACG |
| *Ddx4-Cre* | GGACATGTTCAGGGATCGCCAGGCG | CCATGAGTGAACGAACCTGG |
