# Supplementary material for: GSK-3β protects fetal oocytes from premature death via modulating TAp63 expression in mice
Source: BMC Biol. 2019 Mar 12;17:23. doi: 10.1186/s12915-019-0641-9 (PMC6417224; doi:10.1186/s12915-019-0641-9)
Supplement: Supplementary file 6 — Table S2. ChIP-qPCR primers. (DOCX 11 kb) [file 12915_2019_641_MOESM6_ESM.docx]

**Table S2. ChIP-qPCR primers.**

| ***TAp63* promoter sequence** | **Forward (5’to 3’)** | **Reverse (5’to 3’)** |
| --- | --- | --- |
| -1230~-1120 | CATCTCACAACTAAGAGTAGAC | GCTCATTGGAATCAAGTGTT |
| -963~-793 | GATGTTAGACACGCTCAGT | ATGCCACAGCCATAGACA |
| -783~-627  -631~-510 | TGTGGCATGAATGGAGATAT  GGATTGGTTTACAGCCTACA | CTGAATGTAGGCTGTAAACC  GTCTCATTCTATTATGCCTCAC |
