# Supplementary material for: GSK-3β protects fetal oocytes from premature death via modulating TAp63 expression in mice
Source: BMC Biol. 2019 Mar 12;17:23. doi: 10.1186/s12915-019-0641-9 (PMC6417224; doi:10.1186/s12915-019-0641-9)
Supplement: Supplementary file 7 — Table S3. Antibody information. (DOCX 13 kb) [file 12915_2019_641_MOESM7_ESM.docx]

**Table S3. Antibody information.**

| **Antibody** | **Catalogue** | **Batch No.** | **RRID** | **Manufacturer** |
| --- | --- | --- | --- | --- |
| GSK-3β | #12456P | 1 | AB_2636978 | Cell Signaling Technology |
| DDX4 | #ab27591 | GR290112-1 | AB_11139638 | Abcam |
| cleaved-Caspase-3 | #AC033 |  |  | Beyotime Biotechnology |
| MSY2 | #sc-21316 | L0204 | AB_2217323 | Santa Cruz Biotechnology |
| PCNA | #sc-56 |  | AB_628109 | Santa Cruz Biotechnology |
| BrdU | #G3G4 |  | AB_1157913 | DSHB |
| SYCP3 | #sc-20845 | 10806 | AB_2087203 | Santa Cruz Biotechnology |
| β-catenin | #sc-7199 |  | AB_634603 | Santa Cruz Biotechnology |
| active-β-catenin | #05-665 | 2013029 | AB_309887 | Millipore |
| p-ATM | #AA866-1 |  |  | Beyotime Biotechnology |
| p-CHK2 | #BS4043 | CN883300 | AB_1662753 | Bioworld |
| p63 | #BS1279 | CD46170 | AB_1663924 | Bioworld |
| RAD51 | #sc-8349 | G3012 | AB_2253533 | Santa Cruz Biotechnology |
| γ-H2AX | #NB100-2280 |  | AB_10000580 | NOVUS |
| p-GSK-3β | #AG753-1 |  |  | Beyotime Biotechnology |
| p-β-catenin | #9561S | 12 | AB_331729 | Cell Signaling Technology |
| FOXL2 | #ab5096 |  | AB_304750 | Abcam |
| GAPDH | #AM4300 | 1205023 | AB_437392 | Ambion |
